# DeepMAge: A Methylation Aging Clock Developed with Deep Learning

**DOI:** 10.14336/AD.2020.1202

**Published:** 2021-08-01

**Authors:** Fedor Galkin, Polina Mamoshina, Kirill Kochetov, Denis Sidorenko, Alex Zhavoronkov

**Affiliations:** ^1^Deep Longevity Limited, Hong Kong.; ^2^Integrative Genomics of Ageing Group, Institute of Ageing and Chronic Disease, University of Liverpool, Liverpool, UK.; ^3^Insilico Medicine Hong Kong Limited, Hong Kong Science and Technology Park, Hong Kong.; ^4^Buck Institute for Research on Aging, Novato, CA, USA.

**Keywords:** aging, DNA methylation, epigenetics, artificial intelligence

## Abstract

DNA methylation aging clocks have become an invaluable tool in biogerontology research since their inception in 2013. Today, a variety of machine learning approaches have been tested for the purpose of predicting human age based on molecular-level features. Among these, deep learning, or neural networks, is an especially promising approach that has been used to construct accurate clocks using blood biochemistry, transcriptomics, and microbiomics data—feats unachieved by other algorithms. In this article, we explore how deep learning performs in a DNA methylation setting and compare it to the current industry standard—the 353 CpG clock published in 2013. The aging clock we are presenting (DeepMAge) is a neural network regressor trained on 4,930 blood DNA methylation profiles from 17 studies. Its absolute median error was 2.77 years in an independent verification set of 1,293 samples from 15 studies. DeepMAge shows biological relevance by assigning a higher predicted age to people with various health-related conditions, such as ovarian cancer, irritable bowel diseases, and multiple sclerosis.

## INTRODUCTION

### Disruptive potential of AI solutions in science

Deep learning algorithms are best known for their achievements in text, sound, and image processing. Thanks to their easily understood results and eye-catching demonstrations, neural networks have probably become the best-known machine learning method among laypeople.

Deep learning solutions are frequently dubbed as artificial intelligence (AI). The futuristic, imperative, and slightly menacing connotations of this term feed human imagination, resulting in a plethora of fictional stories featuring AI. Reality and fiction form a feed-forward loop: AI concepts previously considered fantastic become actual feats of science and engineering, which then widens the spectrum of “impossible and fantastic” AI concepts for the next iteration.

Generative models that write comprehensive stories, such as the Generative Pre-trained Transformer 3 language model (GPT-3, https://github.com/openai/gpt-3), self-driving cars, and digital decision-making models used by the military, make enticing news stories [1]. Meanwhile, the AI instances developed for research purposes are much less known to the public. This, however, does not indicate a lack of progress or relevance. On the contrary, state-of-the-art deep learning models in biology, chemistry, and medicine could potentially disrupt the healthcare and pharmaceutical industries.

For example, deep learning models already perform similarly to trained professionals in the differential diagnosis of brain diseases based on magnetic resonance imaging (MRI) scans [2]. The accuracy of the described AI diagnostics tool is equal to that of neuroradiologists (86-91% correct top three differential diagnoses), and it even outperforms less specialized radiologists (57% for general radiologists). In another recent study, a deep learning tool was developed to measure blood flow parameters based on heart MRI scans [3]. The authors established that these measurements determined prognostic value in a cohort of 1,049 cardiology patients followed up for a median of 605 days. An increase of one standard deviation in myocardial blood flow lowered mortality by 36%. Clinical AI systems such as these can be used to significantly reduce exam times and to quantify health risks, ultimately increasing the throughput and cost-effectiveness of a healthcare system. The same models can also be used to analyze academic data, assess treatment efficiency, and establish accurate reference knowledge [4].

In pharmacology, deep learning methods can be employed to streamline drug design. Generative Tensorial Reinforcement Learning (GENTRL)—a deep neural network generator—was used to discover a DDR1 small molecule inhibitor whose *in vivo* beneficial properties were established within three weeks after the experiment was launched [5]. Using traditional iterative design methods, the same target-to-hit stage of drug design would take months or even years.

In biogerontology, deep learning methods have been used to create a number of novel age predictors, which can be used to develop geroprotective interventions or to help aging-conscious people understand and, potentially, affect their pace of aging. For example, age predictions made by a hematological deep aging clock have been shown to be associated with mortality risk [6]. The clock uses standard blood parameters measured during a typical check-up, such as glucose, cholesterol, and platelet count, which could be manipulated with lifestyle changes, dietary and pharmacological interventions. Based on its predictions, people were grouped into normal-agers, over-agers, and under-agers, depending on whether their prediction error was within the ±5-year range. Over-agers had up to double the mortality rate of normal-agers and quadruple that of under-agers.

Similar aging clocks are available for other biodata types, such as transcriptomes and microbiomes [7, 8]. Currently, the only published aging-clock solutions for these data dimensions are in the deep learning family [9]. These models allow researchers to assess potential geroprotective interventions from different angles and parse the entangled concept of organismal aging into discrete packages of a lower level. The tolerance of deep learning methods to non-linear cases instills hope that in the future, even more types of complex biological data will be interpreted within the context of aging. Among other deep learning models, variational autoencoders hold great potential, as they can create digital patients. These human avatars can then be used to emulate the aging process and test geroprotective interventions *in silico* [10, 11].

### DNA methylation (DNAm) aging clock progress

The first aging clocks based on omics data date back to 2013. That year, two seminal articles dedicated to DNAm aging clocks were published: [12] by Horvath and [13] by Hannum et al. Each study describes an algorithm that estimates human chronological age based on data obtained from Illumina DNAm microarrays. Their implementations are different, yet they share a common nature. Both solutions rely on the elastic net regularized regression method, a type of linear model in which the methylation levels at specific dinucleotide CpG loci are assigned weights and then summed to obtain a final prediction.

Horvath’s model includes 353 CpG sites on Illumina 450k and 27k DNAm array platforms, while the model published by Hannum et al. is based on 71 sites on Illumina 450k platforms. Interestingly, the CpG sites used by the two models have little overlap, as only six sites are shared between them. Despite the significant differences in data preprocessing, training samples, and final features, these aging clocks show similar performance when validated in a variety of experimental settings [14]. The error margins reported by their authors are similar as well: a median absolute error (MedAE) of 3.6 years for the 353 CpG clock and a root mean square error (RMSE) of 3.9 years for the 71 CpG clock.

The results published in 2013 inspired many other researchers to develop their own implementations using the same concept. More recent DNAm clocks include [15] by Weidner et al., [16] by Lin et al., and [17] by Levine et al. They show that there are multiple sets of CpG sites that can be used to achieve comparable accuracy [9].

All such aging clocks have proven to be invaluable tools for biogerontology since they offer a unique opportunity to quantify the aging process. This ability is essential for testing geroprotective interventions and studying age-related diseases. Further research has shown significant differences in the ways various DNAm clocks operate, more specifically the low correlation between their predictions and their unequal sensitivity to certain age-related diseases [18].

This profusion of equally good DNAm clock solutions suggests that CpG methylation status is a mathematically degenerate data type. There may be countless non-overlapping combinations of CpG sites that can serve as the basis for an aging clock. Whether all DNAm clocks correspond to the same function of age or fundamentally different processes is an ongoing debate.

In this article, we present another take on DNAm clocks. Our solution, DeepMAge, relies not on a linear regression method but a deep learning approach. Our neural network shows superior accuracy when compared to elastic net solutions, and it shows disease relevance by predicting higher age values for people with various disorders, even when linear models fail to detect any difference. Considering the recent feats of neural networks applied to other biogerontological problems, we hope that DeepMAge can help the community gain a deeper understanding of how the epigenetic landscape shifts over time.

The neural network approach allows the epigenetic dimension of aging to be distilled and integrated with other types of biological information. Models such as DeepMAge can be treated as feature reduction methods that compress large, unrefined vectors into compact latent representations where aging trends are easier to outline. A combination of these representations can be used as an input for a multi-modal aging clock, which will account for multiple aging-related processes. Currently, the bottleneck for developing such models is the shortage of publicly available multi-modal datasets that would contain longitudinal data for multiple aging dimensions: gene expression values, DNA methylation levels, metabolic profiles, or image data [19].

## MATERIALS AND METHODS

### Data availability

This study was carried out using datasets collected from the publicly available Gene Expression Omnibus repository (www.ncbi.nlm.nih.gov/geo/).

Overall, 32 studies were used with 6,411 DNAm profiles in total. Among these, 17 studies and 4,930 samples were included in the training set. The other 15 studies and 1,293 profiles were used in the verification set. Samples annotated as being in the case cohorts of their original studies were explored separately. All metrics for both the verification and training sets were calculated using only the samples marked as control cohorts in the repository.

The exact study identifiers of the training set are: GSE106648, GSE125105, GSE128235, GSE19711, GSE27044, GSE30870, GSE40279, GSE41037, GSE52588, GSE53740, GSE58119, GSE67530, GSE77445, GSE77696, GSE81961, GSE84624, and GSE97362. The exact study identifiers of the verification set are: GSE102177, GSE103911, GSE105123, GSE107459, GSE107737, GSE112696, GSE34639, GSE37008, GSE59065, GSE61496, GSE79329, GSE87582, GSE87640, GSE98876, and GSE99624.

All data used in this study were obtained from blood samples on Infinium Human Methylation 450K and 27K BeadChip platforms (manufactured by Illumina). Only studies with available age metadata and raw files were selected.

### DNAm profile preparation

The data were downloaded as either raw intensities or files in the IDAT format. The lumi R package (v2.38.0) was used for intra-study color correction and normalization [20]. Only 24,538 CpG sites shared between the 450K and 27K platforms were used, minus sex chromosome sites and sites with orthologous sequences on multiple chromosomes.

Approximately 17% of the samples used in this project were associated with integer age values. Such samples have a *de facto* understated chronological age. To avoid introducing this bias into the model, 0.5-year counts were added to the integer ages. No counts were added to the float age values.

### Horvath clock replication

We used the 353 regression coefficients (plus intercept) published in the original paper by Horvath [12] to reconstruct the linear regression model. The model was then used to estimate the logarithmically transformed age, as described in [12].

The reverse transform we used is:

$$\mathrm{Age}=21\times {\mathrm{Exp}}^{\mathrm{Prediction}}-1,\mathrm{if on}=0$$

Age=21×Prediction+20,if on>0

Additionally, a *de novo* elastic net model was trained using a protocol from Horvath [12]. The script we used can be found in the Supplementary Materials section of this article.

To compare the accuracy of DeepMAge to that of the 353 CpG clock, MedAE and mean absolute error (MAE) metrics are used most frequently in this article. Although the original paper for the 353 CpG clock uses mostly MedAE, we included MAE to allow comparison with other aging clocks that have only one of these scores reported. MedAE and MAE are equal to zero when the predicted values of age are in perfect agreement with the actual values.

The formulas for MedAE and MAE are as follows:

$$\mathrm{MedAE}=\mathrm{Median}\left(\left|{\mathrm{Age}}_{\mathrm{true},i}-{\mathrm{Age}}_{\mathrm{predicted},i}\right|\right),\mathrm{foritalic}?\left(1,N\right),\mathrm{where N is the total number of samples}$$

$$\mathrm{MAE}=\frac{1}{N}\left|{\mathrm{Age}}_{\mathrm{true},i}-{\mathrm{Age}}_{\mathrm{predicted},i}\right|,\mathrm{where N is the total number of samples}$$

### Deep learning

We performed age prediction as a regression task in which the model takes DNAm beta vectors as input and then outputs a continuous age value. To allow fitting the data with high dependencies, we used a deep neural network model with multiple hidden layers. In particular, we used feed-forward neural networks with more than three hidden layers.

Due to the high dimensionality of the input (the original data included 24,538 features), feature selection was applied before training of the final model. First, a neural network was trained on the original data, then deep feature selection [21] and gradient-based feature selection methods [22] were applied to find the most important features in terms of impact on model output. To optimize model parameters, we used a grid search over the model depth (from two to five hidden layers), a neuron count per hidden layer (from 128 to 1,024), an activation function (exponential linear unit - ELU, rectified linear unit - ReLU, scaled exponential linear unit - SELU), an optimizing algorithm (Adam, Amsgrad, and Nadam), and a regularization algorithm: dropout [23] (with rate from 0.15 to 0.5) and L2 regularization (with L2 coefficient from 1e-6 to 0.1). Next, the best feature selection method was identified in terms of the target metric, i.e., MAE. Finally, the 1,000 most important features were fixed using an algorithm that calculates the 95^th^ percentile of the gradients moduli based on the model input and input neurons (with corresponding input features), with the greatest gradients modulus being the most important [22].

The final model was trained using the 1,000 most important features. To optimize model parameters, we used a grid search with the same grid parameters as in the previous search. We minimized the MAE loss function using a backpropagation algorithm. After the optimization procedure, the best model had the ELU function applied after each layer; Adam as the optimizer of the cost function with a learning rate of 10^­4^; a 30% dropout probability at each layer; and L2 regularization with a 10^-3^ coefficient. The final best neural network model consisted of four hidden layers with 512 neurons each.

We trained the networks with fivefold cross-validation (CV) to compensate for overfitting and to receive more robust performance metrics in both cases: feature selection and the final model. The Python version of the Keras library (https://keras.io/) with TensorFlow (www.tensorflow.org/) backend for neural network implementation was used. All experiments were conducted using an NVIDIA GeForce 1080Ti graphics processing unit.

### Statistical analysis

The accuracy metrics for model performance included MAE, MedAE, Pearson’s r, RMSE, and coefficient of determination (R^2^). These metrics were calculated using the Python 3.6 sklearn.metrics (v.0.22.1; https://scikit-learn.org) and scipy.stats packages (v.1.4.1; www.scipy.org/).

The Mann-Whitney U test (MW test) for estimating the significance of differences in sample means was imported from the scipy.stats package (v.1.4.1; www.scipy.org/).

Pathway enrichment was performed using the Gene Ontology web resource (http://geneontology.org/) [24].

To estimate the effect of body mass index (BMI) on age prediction, the Python statsmodels.regression. linear_model.OLS class from statsmodels (v0.11.0; www.statsmodels.org) was used.

Data visualization was conducted with Plotly (v.4.5.0; https://plotly.com/) for Python and Seaborn (v.0.10.0; https://seaborn.pydata.org).

**Table 1:** Accuracy metrics for DeepMAge. The accuracy achieved in cross-validation (CV column, MedAE = 2.24 years) was only slightly reduced during verification (healthy verification column, MedAE = 2.77 years). Accuracy declined in the samples with various health-related conditions (case verification column, MedAE = 4.35 years).

	CV	Healthy verification	Case training	Case verification
MedAE, years	2.24	2.77	3.29	4.18
MAE, years	3.21	3.80	4.74	5.08
R^2^	0.96	0.93	0.88	0.82
Pearson’s r	0.98	0.97	0.94	0.94
RMSE, years	4.55	5.44	7.51	6.24
N	4,930	1,293	1,093	439

CV = Cross-validation; MAE = Mean absolute error; MedAE = Median absolute error; R^2^ = Coefficient of determination; RMSE = Root mean square error; N = Number of samples in the subsample

## RESULTS

### DeepMAge performance in healthy and ill individuals

**Figure 1.**
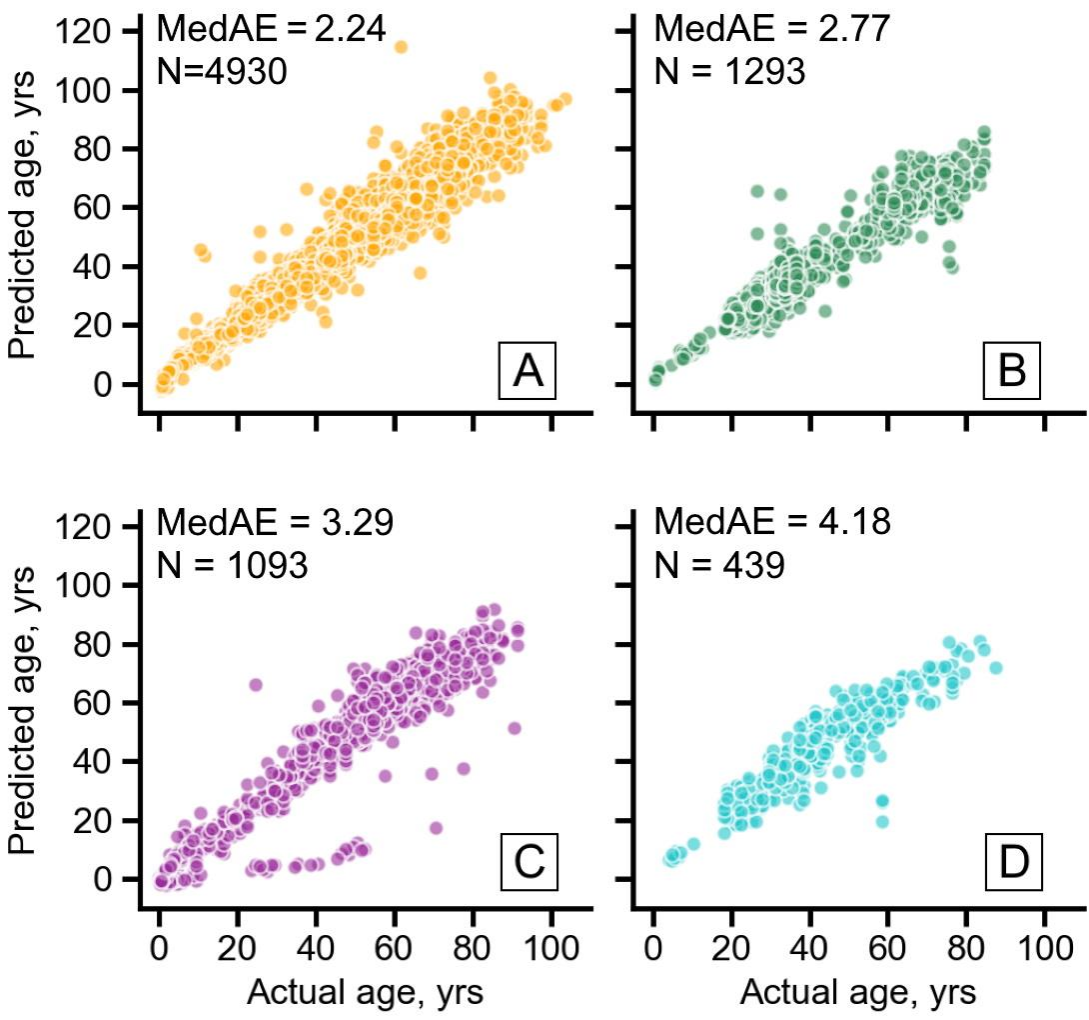
Scatter plot of DeepMAge predictions in 4 data cohorts”. DeepMAge accurately predicted the chronological age of healthy people from the training set (A), healthy people from the verification set (B), and remained accurate in the aggregations of case cohorts from the studies included in the training set (C) and the verification set (D). Scatter plot in panel A shows the per-fold predictions obtained during CV, and the other panels show the predictions by the final model. MedAE = Median absolute error measured in years, N = Number of samples in a corresponding cohort (see Supplementary Figures 1-3 for a more detailed visualization).

**Figure 2.**
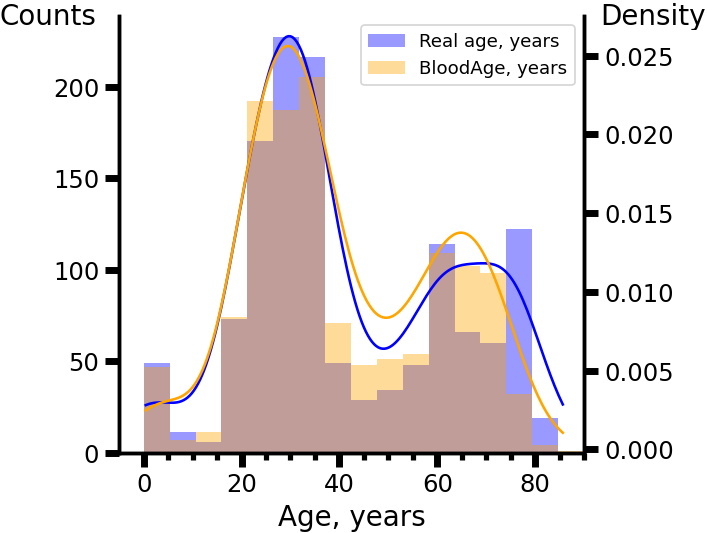
The DeepMAge prediction age distribution in the verification set closely resembled the real age distribution. Distributions were obtained using Gaussian kernel with 0.3σ bandwidth, where σ is the standard deviation of the age values.

We trained the deep neural network DeepMAge using a collection of 4,930 blood DNAm profiles, obtaining a cross-validated MedAE of 2.24 years (Table 1) from the control cohorts of 17 studies (Supplementary Table 1). The results of DeepMAge testing on control cohorts from 15 independent datasets (1,293 samples) were slightly less accurate, with a MedAE of 2.77 years (Fig. 1, Supplementary Fig. 1-3). The prediction distribution for samples from the verification set (except for people over 70 years old) closely resembled the actual age distribution (Fig. 2).

Most surprisingly, the DeepMAge predictions for the aggregated case cohorts were almost as accurate as for the healthy cohort. Case cohorts from the studies used in the training sample displayed a MedAE of 3.29 years, while the MedAE for the case cohorts in the verification sample was 4.18 years (Fig. 1 and Supplementary Fig. 2).

No significant differences between male and female absolute error distributions were detected with an MW test on the total samples. When age groups from the verification set were tested separately, significant sex-related differences in the 55-65 and 65-75 age groups were detected (Table 2 and Supplementary Fig. 4). The mean errors found for women in these age ranges were higher (*p-value* < 0.05), while the ages of 65-75-year-old women were predicted almost 2 years more accurately in absolute terms (*p-value* < 0.01). These findings in the verification set went against the error distributions in the training set and thus were probably due to sample bias rather than any biologically significant factors.

We then further inspected the specific studies with a case-control setting. Comparing the average prediction errors of the case and control cohorts, DeepMAge reacted only to certain conditions (Table 3). Out of 12 such studies, only five showed significantly elevated prediction errors for the case cohorts. In the study on tauopathic frontotemporal dementia and palsy, cases were 1.00 years older than controls. People with inflammatory bowel diseases (IBD) were predicted by DeepMAge to be 1.23 years older than controls. Women with ovarian cancer were predicted to be 1.70 years older. Multiple sclerosis patients were predicted to be 2.10 years older. People with congenital CHARGE and Kabuki syndromes were quite interestingly predicted to be 5.28 years younger than controls. Congenital hypopituitarism was associated with predictions 5.64 years older than predictions for controls. These results may indicate a faster pace of aging in people with these pathologies (except for CHARGE and Kabuki syndromes).

**Table 2:** DeepMAge prediction errors are not significantly different for younger males and females. Sex-related differences in age prediction for older adults are inconsistent between the CV and the verification sets.

Set	Error, years					Absolute Error, years						N
	Years	(20-45)	(45-55)	(55-65)	(65-75)	(20-75)	(20-45)	(45-55)	(55-65)	(65-75)	(20-75)	
Verification	Male	+0.48	-2.50	-1.46*	-4.76*	-0.87*	+2.97	+4.04	+3.98	+6.04*	+3.68	574
	Female	+0.23	-3.58	-0.06*	-1.78*	-0.12*	+3.24	+4.48	+3.50	+4.13*	+3.40	494
	N	707	62	163	136	1068	707	62	163	136	1068	
CV	Male	+0.62	+2.14*	+0.62*	+0.81*	0.97*	+2.84	+3.80	+4.00	+4.89	+3.53	1452
	Female	+0.65	+0.41*	-0.54 *	-2.17*	-0.34*	+2.76	+3.59	+3.77	+4.58	+3.59	2058
	N	1323	670	897	620	3510	1323	670	897	620	3510	

The significant differences are marked with “*” (*p-value* < 0.05 in the MW test). CV = Cross-validation; MW = Mann-Whitney U test; N = Number of samples in the age range or sex subsample.

### Comparison to the 353 CpG aging clock

To gain more insight into whether deep learning offers any benefit compared to linear models, we used the published 353 CpG clock to predict age for the datasets we used in this project. The accuracy reported in its original publication [12] is a MedAE of 3.56 years, which is close to its reproduced accuracy in our data collection (MedAE = 3.51 years, Table 4). In this respect, DeepMAge significantly outperformed the 353 CpG clock with a MedAE of 2.24 years during CV and 2.77 years during verification (Table 1).

The correlation between predictions by the 353 CpG clock and DeepMAge for the verification set was significantly high (Pearson’s r = 0.96) for the healthy verification cohort (1,293 samples). The same was observed in the samples from the case cohorts within the training studies set (Pearson’s r = 0.96, 1,093 donors).

Two studies used in our verification cohort were also used for verification in the original 353 CpG clock publication: GSE34639 (48 samples) and GSE37008 (99 samples). In these two studies, the 353 CpG clock showed superior performance compared to DeepMAge (Table 4). We then examined the other verification datasets we had, which were not used in the original paper by Horvath. Overall, in seven out of the 15 datasets we compared, DeepMAge showed superior performance according to both MedAE and Pearson’s r. In 13 out of 15 studies DeepMAge performed better according to at least one metric used (Table 4).

**Figure 3.**
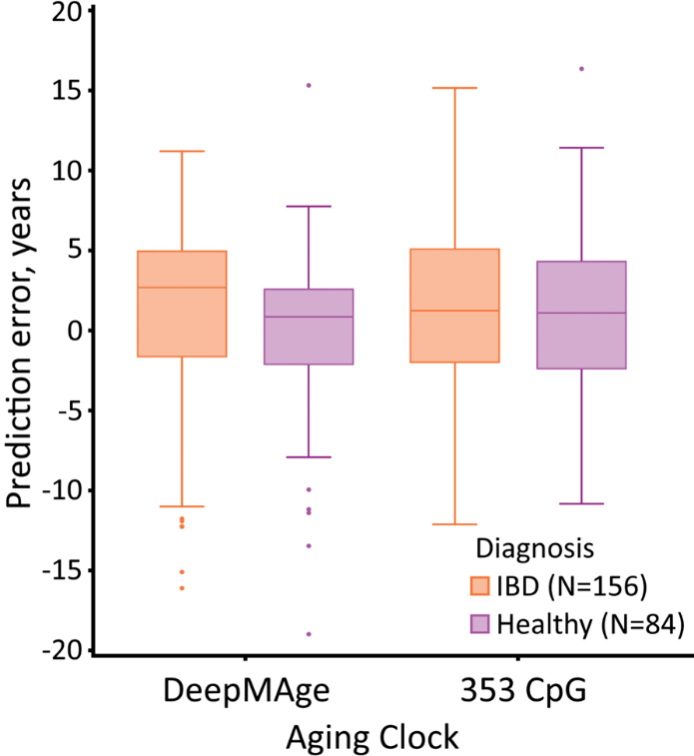
DeepMAge, but not the 353 CpG clock, predicted donors with IBD (GEO study accession GSE87640) to be on average 1.23 years older than the healthy donors from the same study (*p-value* = 1.24E­3). Outliers outside the (-20; +20) prediction error window were removed from the image; The box is formed by the interquartile range with the median marked inside it. Whiskers protrude no farther than 1.5 times the interquartile range. GEO = Gene Expression Omnibus; IBD = Inflammatory bowel disease; N= Number of samples in a corresponding cohort.

In certain cases, DeepMAge was more sensitive to donor conditions than the 353 CpG clock. For example, GSE87640 contains healthy donors (84) and donors with IBD (156 donors), namely ulcerative colitis and Crohn’s disease. DeepMAge predicted the IBD cohort to be significantly older (*p-value* < 0.001) than the healthy cohort, with the delta being 1.2-1.8 years, depending on whether the mean or median error is used (Fig. 3 and Table 3). This difference was not observed in the 353 CpG clock predictions (delta MAE = 0.3 years, *p-value* = 0.21).

**Table 3:** Five diseases (including ovarian cancer and multiple sclerosis) were associated with significantly higher age predictions (*p-value* (MW) < 0.05).

GEO ID	Mean error in control	Mean error in cases	*p-value* (MW)	*p-value* (random MW)	N control	N case	N total	DeepMAge sample	Case cohort description
GSE53740*	-0.37	+0.63	2.70E-2	1.50E-1	197	186	383	Training	Neurodegenerative tauopathy
GSE19711*	-2.97	-1.27	9.84E-6	4.39E-1	272	264	536	Training	Ovarian cancer
GSE77696	+4.43	+3.96	1.31E-1	5.29E-2	117	261	378	Training	HIV
GSE106648*	-1.84	+0.26	2.17E-8	2.52E-1	139	140	279	Training	Multiple sclerosis
GSE67530	-2.66	-1.63	1.12E-1	1.01E-1	105	39	144	Training	Acute respiratory distress syndrome
GSE52588	0.67	1.19	1.71E-1	4.84E-1	58	29	87	Training	Down syndrome
GSE97362*	1.24	-4.04	2.05E-3	9.30E-2	83	150	233	Training	CHARGE / Kabuki syndrome
GSE84624	0.54	0.73	4.39E-1	9.87E-2	24	24	48	Training	Kawasaki disease
GSE112696	4.24	4.56	3.44E-1	1.89E-1	6	6	12	Verification	Myasthenia gravis
GSE102177	1.99	1.91	4.94E-1	2.38E-1	18	18	36	Verification	Maternal gestational diabetes
GSE87582	-9.59	-3.79	1.08E-1	2.82E-1	1	20	21	Verification	HIV
GSE107737*	-1.98	3.66	3.63E-3	1.56E-1	12	12	24	Verification	Congenital hypopituitarism
GSE87640*	-0.20	1.03	1.24E-3	3.57E-1	84	156	240	Verification	Inflammatory bowel diseases
GSE99624	-1.58	-3.99	6.43E-2	3.76E-1	16	32	48	Verification	Ostheoporosis

*p­value* (MW) is the significance of the MW test for equal mean prediction error between the case and control cohorts in each study; “*” marks the studies with a significant (*p-value*<0.05) MW test result; *p-value*(random MW) is the significance of the test for a permuted sample. For the control samples marked as “Training,” the predictions were obtained during CV; for the case samples marked as “Training,” the predictions were obtained with the final model, which had not been previously exposed to these samples. The studies in which the studied condition was significantly associated with higher DeepMAge predictions are marked in green. CV = Cross-validation; GEO ID = Gene Expression Omnibus accession number; HIV = Human Immunodeficiency Virus; MW = Mann-Whitney U test; N = Number of samples in the corresponding GEO project cohorts.

DeepMAge predictions were significantly less affected by the sex of the donors. DeepMAge showed no significant difference between predictions for males and females in the total verification cohort (0-91 years, also see Table 2 for age group-specific differences in DeepMAge performance). Meanwhile, the 353 CpG aging clock predicted males to be on average 1.42 years older than females (*p-value* = 1.2E-8).

We also compared the 353 CpG aging clock and DeepMAge in the context of the effect of obesity on aging. For this task, we used data from GSE37008, which contained 94 individuals with a wide range of BMI values (from 16.17 to 36.26 kg/m^2^). We used ordinary least squares regression to see if the effect of BMI on the predicted age was significant. Age, predicted age, and BMI were scaled to fit a linear model: *[Prediction ~ Actual_Age + Is_Male + BMI]* (Supplementary Fig. 5). The BMI regression coefficient for DeepMAge predictions was positive, with a *p-value* = 0.048. Meanwhile, the positive coefficient for the 353 CpG aging clock had a *p-value* = 0.19 and was much less likely to significantly affect age prediction. This difference in sensitivity toward BMI may indicate that DeepMAge recognizes increased body weight as an aging factor. It should be noted, however, that neither the 353 CpG aging clock nor DeepMAge showed a significant BMI effect in another dataset with 107 individuals—GSE105123. However, this may be attributed to the much narrower range of BMI values in that study: from 19.8 to 25.1 kg/m^2^.

### DeepMAge CpG pathway analysis

**Figure 4.**
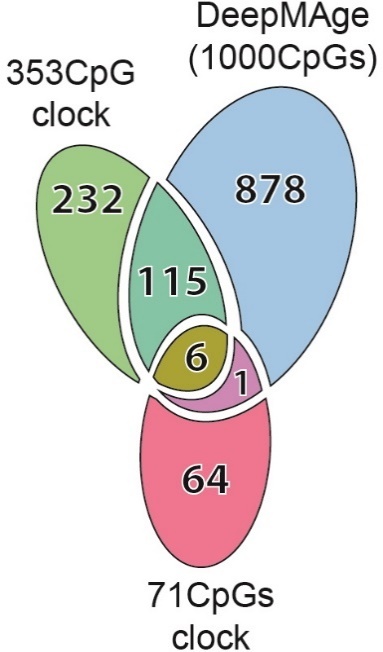
The DeepMAge clock shares 122 CpGs with the 353 CpG clock and seven CpGs with the 71 CpG clock. The latter two were published in 2013.

DeepMAge uses a set of 1,000 CpG sites, of which 121 are shared with the 353 CpG clock and seven are shared with the 71 CpG clock (Fig. 4 and Supplementary Table 2).

We inspected the genes covered by the DeepMAge CpG sites to see if the selected features were enriched in specific pathways. In a Gene Ontology biological function annotation, 289 terms were identified as significantly enriched (FDR < 0.01). The most abundant terms included generic regulatory and signaling terms.

More interestingly, among the 289 enriched terms, 146 are related to tissue development and organ morphogenesis; 57 to neural development, neurogenesis, and synaptic signaling; 14 to circulatory system development and function; 14 to cell differentiation and proliferation (including that of stem cells); 10 to cross-membrane ion transport; nine to cell motility; nine to transcription; and five to locomotion. The top 10 most significantly enriched terms (*p-value* < 1.76E-14) include four terms related to neural function and five terms related to organism development (Supplementary Table 3).

## DISCUSSION

In this article, we present, to the best of our knowledge, the first deep learning DNAm aging clock—DeepMAge—and compare it to Horvath’s 353 CpG aging clock. DeepMAge can estimate human age with a MedAE of 2.77 years, as demonstrated in a verification set containing 1,293 samples. We found that DeepMAge is more accurate in predicting the age of healthy individuals than the 353 CpG clock, which had a MedAE of 3.51 years for the same dataset. DeepMAge is also more accurate than another 71 CpG DNAm aging clock (Supplementary Table 4, see “Comparing DeepMAge to the 71 CpG clock” in the Supplementary Materials section of this article).

Having obtained the deep learning age predictor, we explored its biological relevance in several settings. DeepMAge produced significantly higher age predictions (by 1.23 years on average) for people with IBD compared to healthy people. This difference was not observed in the 353 CpG clock predictions. Some other diseases that may be expected to affect the pace of aging produced similar results (e.g., multiple sclerosis and ovarian cancer) (Table 2). Using a dataset from our verification cohort, we also established higher BMI as a factor contributing to higher predicted age (Supplementary Fig. 5), a finding not supported by the 353 CpG clock.

The sex-related differences in DeepMAge prediction error distributions (Table 2 and Supplementary Fig. 4) were only significant for the older age brackets — 55-65 and 65-75 years — in which women were predicted to be older than men. It was originally hypothesized that sex-specific aging processes, and especially age-related hormonal changes, might affect DeepMAge error distribution. However, the described error patterns are not consistent between the training and verification sets and thus are probably caused by sample bias rather than biological reality.

The 1,000 features that constitute DeepMAge contain 121 CpG sites from the original 353 CpG clock and 75 from the one we reproduced *de novo* (see Supplementary Materials, Supplementary Tables 2, 5). The genes where the DeepMAge CpGs are located are enriched with those taking part in developmental (especially cardio- and neurodevelopmental) processes (Supplementary Table 3). We hypothesize that this may be explained by the antagonistic pleiotropy theory of aging [25]. According to this theory, genes required for the earlier stages of development may sustain their activity beyond their appropriate period of expression. This non-specific activity harms the organism and leads to multiple downstream after-effects that ultimately manifest as aging. Understanding the interactions in which the identified genes take part during the aging process can be useful for rejuvenation research [26].

**Table 4:** In seven out of 15 verification studies, DeepMAge performed better than the 353 CpG clock according to two quality metrics (MedAE and Pearson’s r).

GEO ID	MedAE, years		Pearson’s r		N	Age range, years	Male ratio, %
	DeepMAge	353 CpG	DeepMAge	353 CpG			
GSE107459 **	1.63	3.43	0.79	0.68	127	18-35	0
GSE102177 *	1.87	1.33	0.86	0.83	18	4-14	56
GSE34639 *	1.92	0.22	0.89	0.88	48	0-1	33
GSE105123 **	2.06	2.87	0.47	0.38	107	19-23	58
GSE61496 **	2.14	3.42	0.97	0.95	310	30-74	53
GSE87640 *	2.52	3.02	0.86	0.87	84	20-58	62
GSE98876 **	2.54	4.77	0.89	0.81	71	26-69	100
GSE79329 **	2.63	3.74	0.92	0.89	34	43-70	100
GSE99624 **	2.72	3.73	0.93	0.81	16	49-82	38
GSE107737 *	3.03	3.62	0.34	0.46	12	18-29	100
GSE37008	3.74	2.26	0.81	0.81	99	24-45	37
GSE112696 *	3.75	2.78	0.34	0.23	6	22-27	67
GSE59065 **	4.35	5.01	0.95	0.94	295	22-84	48
GSE103911 *	6.96	6.14	0.85	0.76	65	27-77	71
GSE87582	9.59	6.41	-	-	1	60	100
Average	2.77	3.51	0.97	0.93	1293	0-84	52

There were only two studies for which DeepMAge was not superior to the 353 CpG clock according to at least one metric. Considering the 15 studies in aggregate, DeepMAge provided superior prediction accuracy. “**” marks the studies in which DeepMAge shows superior performance based on both MedAE and Pearson’s r, “*” marks the studies in which DeepMAge shows superior performance based on only one metric. GEO ID = Gene Expression Omnibus accession number; MedAE = Median absolute error; N = Number of samples in the corresponding GEO project.

It should be noted that as part of our data preprocessing pipeline, we added 0.5-year counts to all the specified ages when only full years of age were known. If a person is specified to be 25 years old, their exact age can be anywhere in the (25 years; 26years-1day) range. Thus, using only full years for age is an instance of right censoring, albeit very slight. Since 17% of our data collection contained integer-value ages, the issue of censoring was considered significant, and the counts were introduced.

One may quite reasonably argue that these counts affect the accuracy of the 353 CpG clock to which DeepMAge is compared. In fact, they do. However, the 353 CpG clock becomes less accurate when the counts are removed (MedAE = 3.58 instead of 3.51 years), and a decision was made to keep the counts when working with the 353 CpG clock.

It may also be argued that since DeepMAge and the 353 CpG clock were trained with different data collections, it is incorrect to compare them. To overcome this limitation, we trained an elastic net model following Horvath’s protocol using our data collection to see if a linear model created from scratch could outperform DeepMAge (see Supplementary Materials, Supplementary Fig. 6). This *de novo* elastic net displayed a MedAE of 3.23 years in the verification set, which is more accurate than the original 353 CpG clock (MedAE of 3.51 years) but less accurate than DeepMAge (MedAE of 2.77 years).

We propose DeepMAge as the first deep learning DNAm aging clock that performs better than linear models in certain aspects. Although the biological relevance of DeepMAge has been determined in multiple case-control independent datasets, its mortality association is yet to be established. Newer linear models, such as the 2019 GrimAge trained with the primary purpose of predicting time-to-death, can provide outstanding performance for their prioritized tasks [27]. However, even the newer aging models are scarce in deep learning solutions, although there are enough grounds to suspect that the deep learning approach can improve their already impressive results [9].

Other limitations of DeepMAge include being trained on blood DNAm profiles only. Including training data from other tissues is likely to reduce the overall accuracy of the aging clock, especially if the tissue subsamples are unbalanced. Enabling multi-tissue functionality may require retraining the model. It should also be noted that DeepMAge has not been described in longitudinal or replicate settings to study its prediction stability within the same subject.

Nonetheless, considering the many benefits deep learning algorithms can offer, we hope this study is followed by others. Neural networks can be used to explore individual DNAm landscapes in the context of aging, and they can potentially be used to estimate the risk of certain age-related events in the future given a single observation. Other uses may include aggregating multiple sources of age-related information, including DNAm profiles, to gain a systemic view of the aging process of an individual.

## Conclusions

Epigenetic aging has numerous quantitative models. We have developed DeepMAge — the first deep learning DNAm aging clock, which significantly outperforms more widespread linear models in multiple settings. DeepMAge is sensitive to diseases — a property that makes it valuable as a potential health marker. Further research is required to study DeepMAge reproducibility and robustness in independent longitudinal studies. The approach used to train DeepMAge may be extended to enable more neural network-specific algorithms and digitally simulate the epigenetic aging processes.

## Supplementary Materials

The Supplemenantry data can be found online at: www.aginganddisease.org/EN/10.14336/AD.2020.1202. Additional supplementary materials can be found at https://dx.doi.org/10.17605/OSF.IO/74ZGA.


