# Supplementary material for: DeepMAge: A Methylation Aging Clock Developed with Deep Learning
Source: Aging Dis. 2021 Aug 1;12(5):1252–62. doi: 10.14336/AD.2020.1202 (PMC8279523; doi:10.14336/AD.2020.1202)
Supplement: Supplementary file 1 [file AD-12-5-1252-s.pdf]

DeepMAge: A Methylation Aging Clock Developed with Deep Learning

Fedor Galkin[1,2], Polina Mamoshina[1], Kirill Kochetov[1], Denis Sidorenko[3], Alex Zhavoronkov[1,3,4*]

© 2020. Galkin F et al. Published online at http://www.aginganddisease.org/EN/10.14336/AD.2020.1202

Supplementary Materials

Comparing DeepMAge to a de novo linear model

While Horvath's 353 CpG aging clock is a well-known frame of reference for age predictors, it is not sufficient to show the extra benefits offered by deep learning compared to linear machine learning techniques. Horvath's DNAm clock was trained on a different data collection, and the original paper suggests training models from scratch for new datasets.

Thus, to show deep learning superiority relative to other algorithms, we reproduced an elastic net aging clock as described in Horvath's original paper, using the same data as for DeepMAge. The resulting model contains 348 CpGs, 75 of which overlap with the 353 CpGs originally described by Horvath (Supplementary Table 5). We then verified the obtained linear predictor in the verification set containing 1,293 samples and found that both its MAE = 4.24 and MedAE = 3.23 years were higher than those of DeepMAge in the same set (MAE =3.80, MedAE = 2.77 years). The difference between MAEs, although slight, was deemed significant, with a *p-value* = 0.0001 (Supplementary Fig. 6).

The R script used to reproduce Horvath's method and the corresponding prediction tables can be found in the Supplementary Script section (Supplementary Script 1).

Comparing DeepMAge to the 71 CpG clock

In addition to the 353 CpG clock, we also compared DeepMAge to the 71 CpG and 89 CpG blood DNAm clocks published by Hannum in 2013 [13]. The 71 CpG aging clock was developed using only blood-derived data obtained with the Illumina 450k platform.

We used the coefficients from the original linear regressions and tested these models on studies within the verification set, as defined by the current study. In a set of 1,404 DNAm profiles, both DeepMAge and the 71 CpG clock performed better than the baseline mean age assignment, but DeepMAge showed superior performance in terms of both RMSE and MAE (Supplementary Table 4). The RMSE of the 71 CpG clock (15.12 years) was significantly higher than the accuracy reported in the original paper (3.9 years).

The MedAE achieved by the CpG clock for this set was 7.63 years. The MedAE achieved by DeepMAge for this set was 3.97 years.

The cause of such poor performance of the 71 CpG clock may be its training set size, which contained just 656 individuals. The 353 CpG clock, in turn, was trained on a set containing 7,844 multi-tissue DNAm profiles, and DeepMAge was trained on 4,930 blood samples.

Note: All supplementary tables and the supplementary R script have been uploaded to the Open Science Framework (https://dx.doi.org/10.17605/OSF.IO/74ZGA).

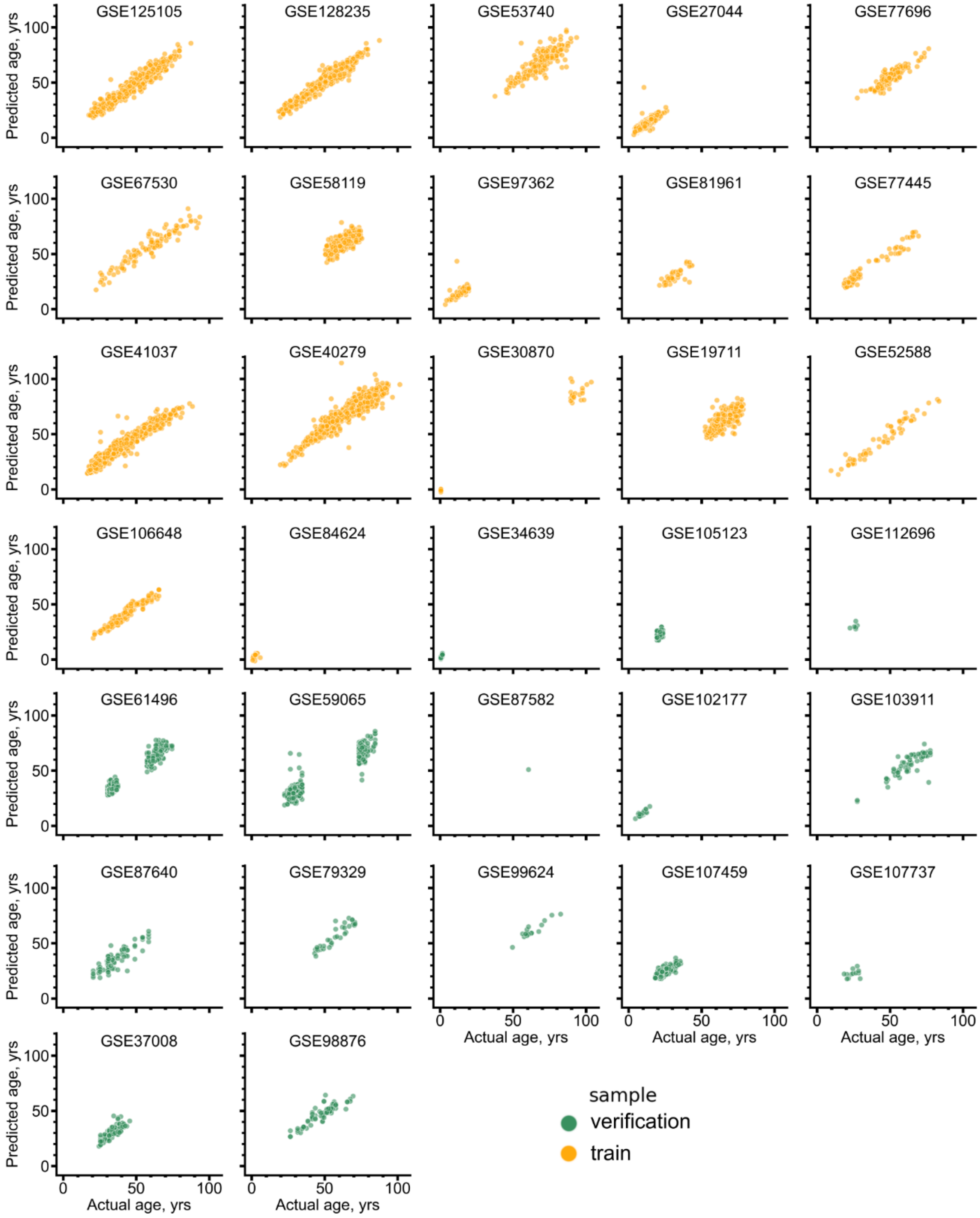

Supplementary Figure 1. DeepMAge accurately predicted the ages of the healthy blood sample donors of all studies from the training and verification sets. Training set predictions were obtained during cross-validation.
Yrs = Years

© 2020. Galkin F et al. Published online at http://www.aginganddisease.org/EN/10.14336/AD.2020.1202

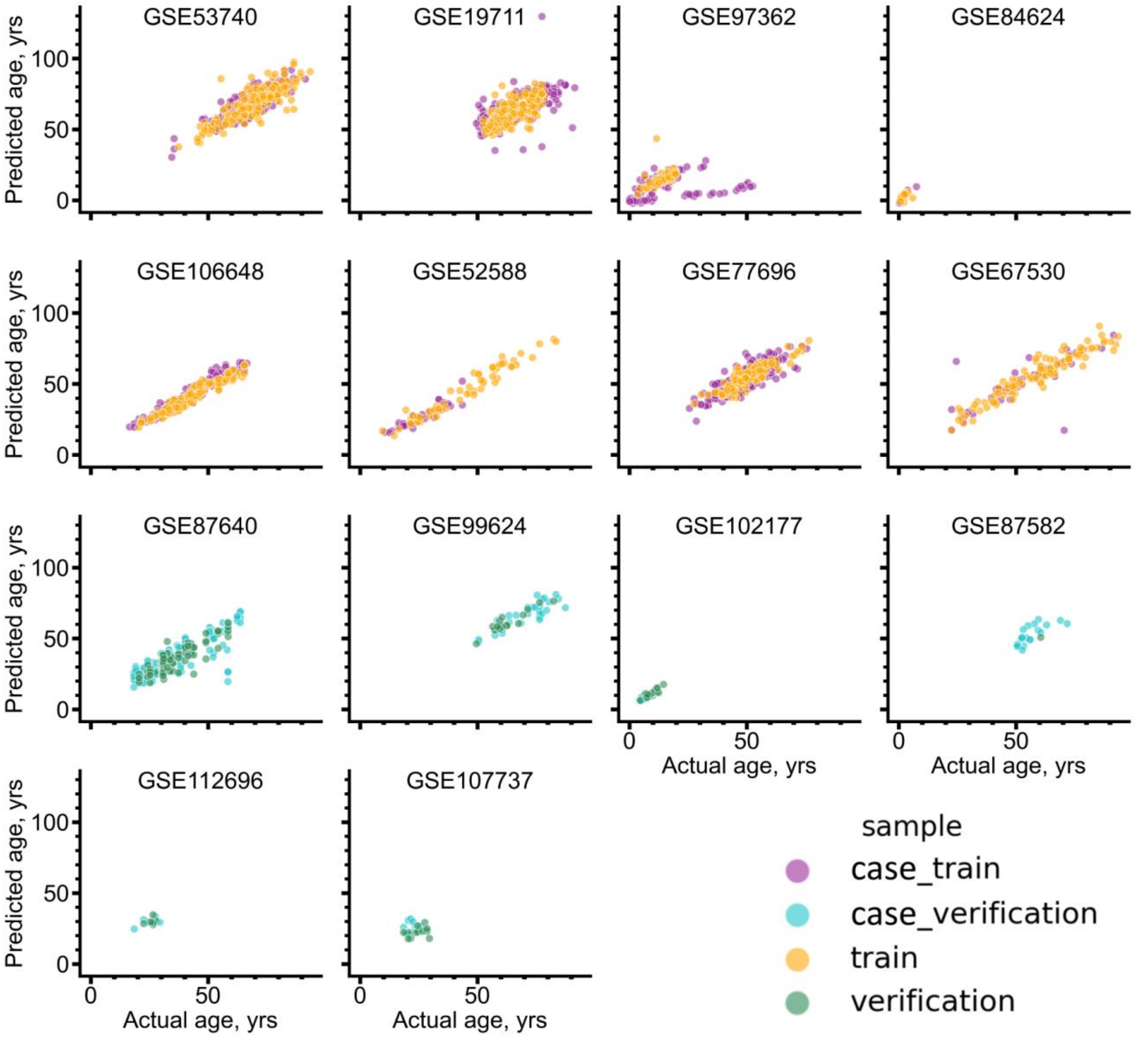

Supplementary Figure 2. DeepMAge accurately predicted the ages of the blood sample donors of the case-control studies from the training and verification sets.
Yrs = Years

© 2020. Galkin F et al. Published online at http://www.aginganddisease.org/EN/10.14336/AD.2020.1202

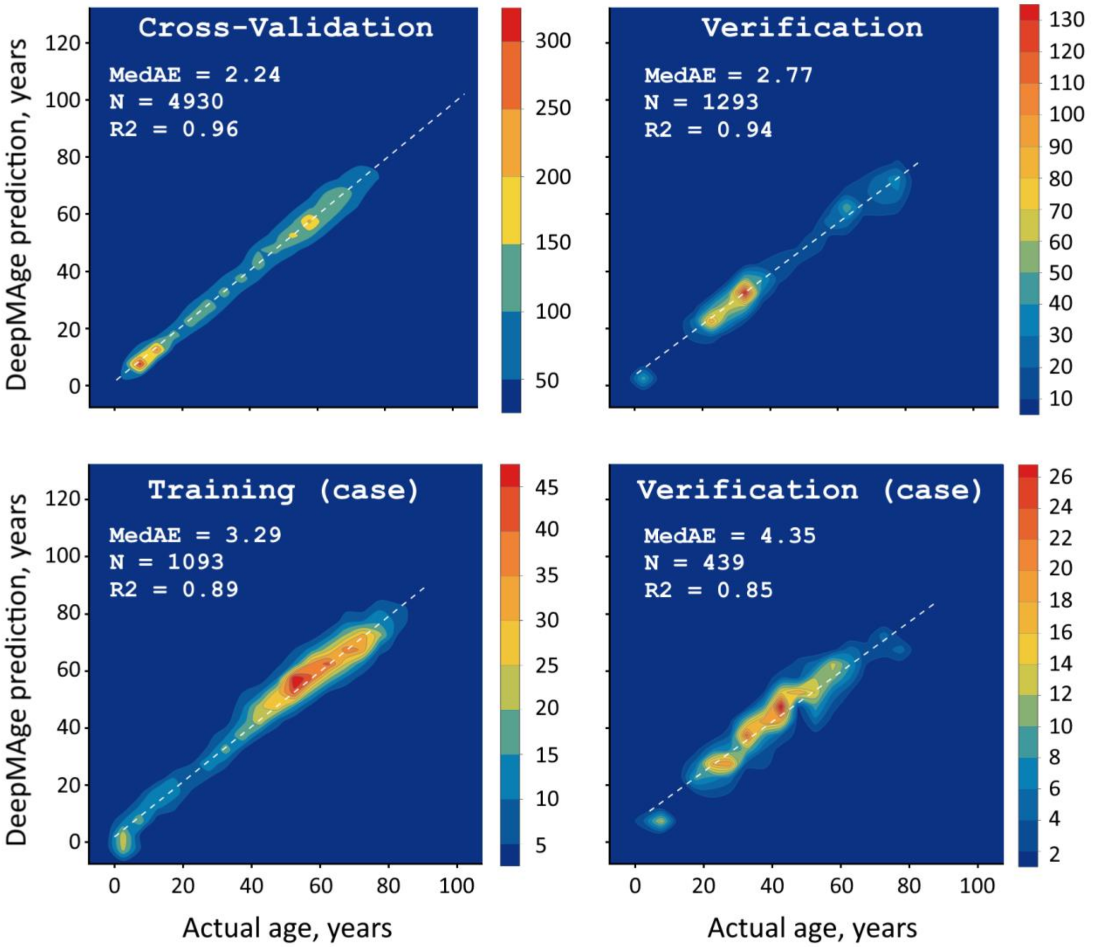

Supplementary Figure 3. KDE representation of Fig.1 in the main text. DeepMAge accurately predicted the chronological ages of both healthy individuals (top) and in an aggregation of case cohorts from multiple studies (bottom). All the cohorts, apart from the "Cross-Validation" cohort, were predicted by the final model. The "Training (case)" cohort refers to the samples that were present in the studies used for training but were excluded from CV because they came from unhealthy donors. Similarly, the "Verification" cohort contains only healthy donors, and the "Verification (case)" cohort contains donors with various health conditions from the same studies. MedAE = Median absolute error measured in years; N = Number of samples in a corresponding cohort; R2 = Coefficient of determination. The white dashed lines are the least squares regressions. The number of counts contained within each contour of the 2D histograms is color-coded according to the color bars to the right of each subplot.

© 2020. Galkin F et al. Published online at http://www.aginganddisease.org/EN/10.14336/AD.2020.1202

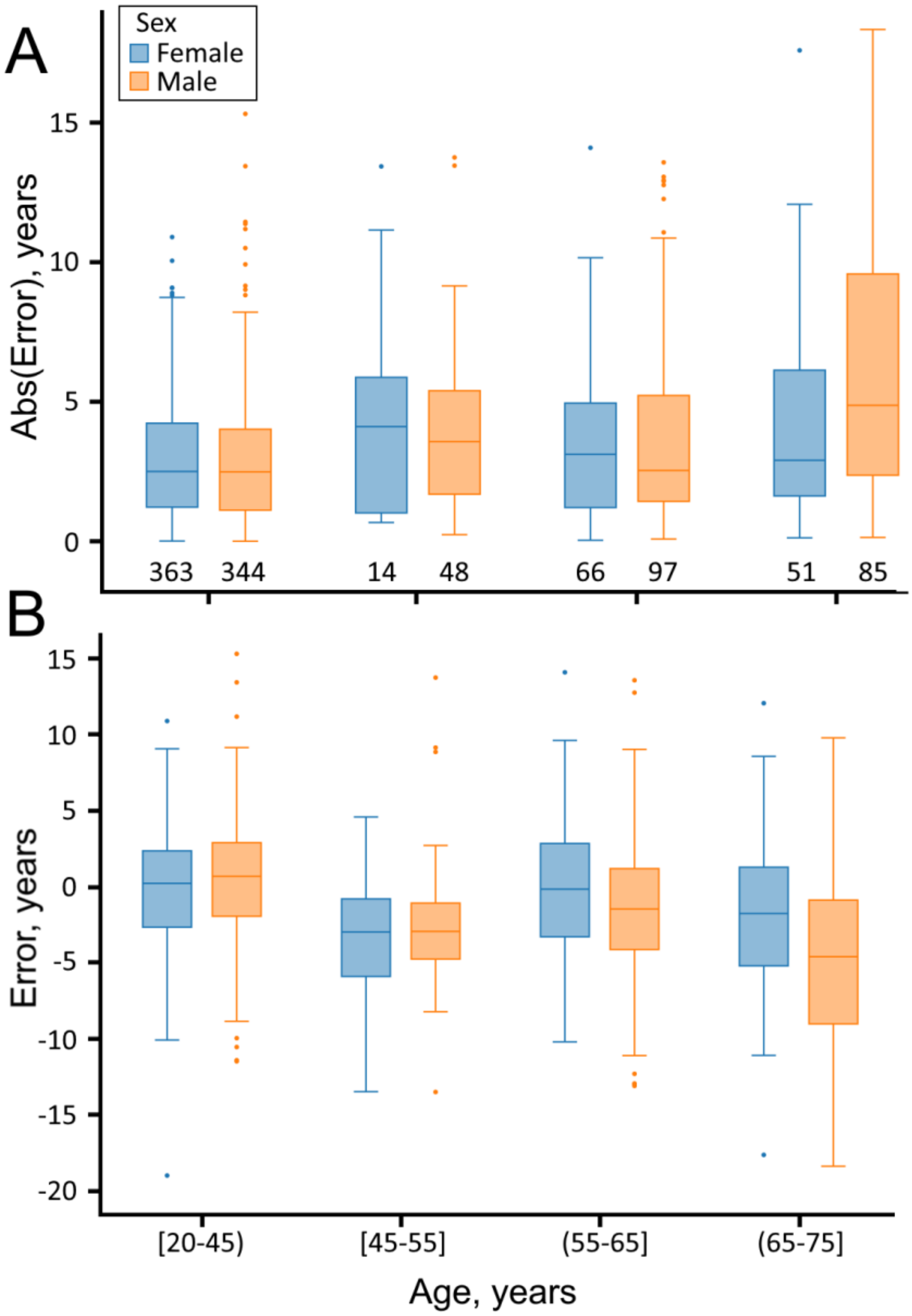

Supplementary Figure 4. Older adults but not younger adults have significantly different DeepMAge error distributions for males and females (Table 2). (A) DeepMAge absolute prediction error per age group in the verification set. The number of samples in each age group is marked below the boxes; **(B)** DeepMAge prediction error per age group in the verification set; The middle lines represent the medians; the box edges represent the quartiles; the whiskers reach out no farther than 1.5 times the interquartile distance. Four upper outliers in the [20-45) female age group were removed for presentation purposes. Abs (Error) = absolute error

© 2020. Galkin F et al. Published online at http://www.aginganddisease.org/EN/10.14336/AD.2020.1202

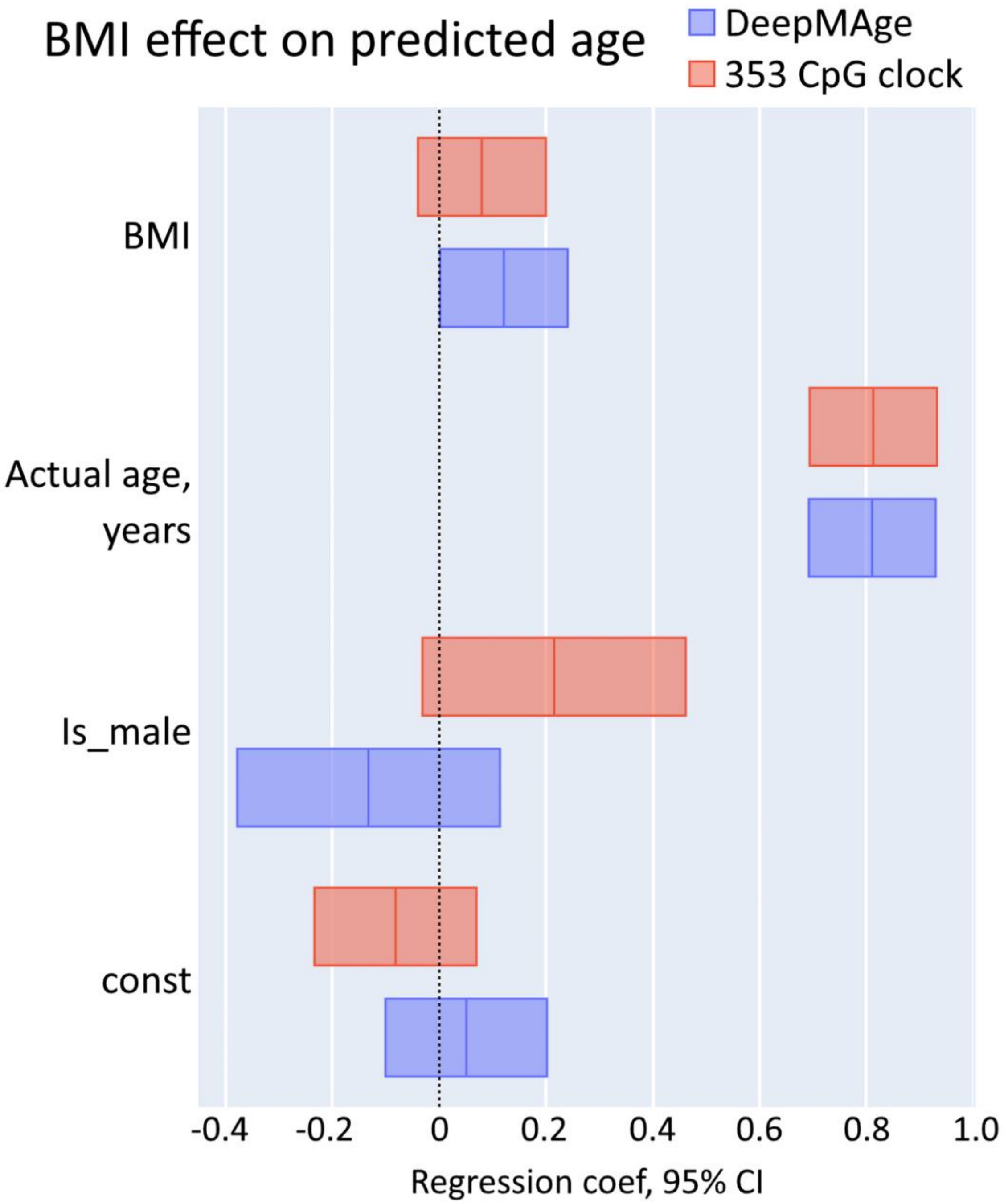

Supplementary Figure 5. Scaled BMI effect on age prediction, as observed in [Predicted ~ Real Age + Sex + BMI] ordinary least squares linear regression. BMI had a significant effect (*p-value* = 0.048) on the predicted age for DeepMAge, but not for Horvath's aging clock (*p-value* = 0.19). Data set used: GSE37008. The middle lines of the boxes represent the point estimate for the regression coefficients; their edges represent the 2.5% and the 97.5% CI borders. BMI = Body mass index; CI = Confidence interval; const = Model intercept

© 2020. Galkin G et al. Published online at http://www.aginganddisease.org/EN/10.14336/AD.2020.1202

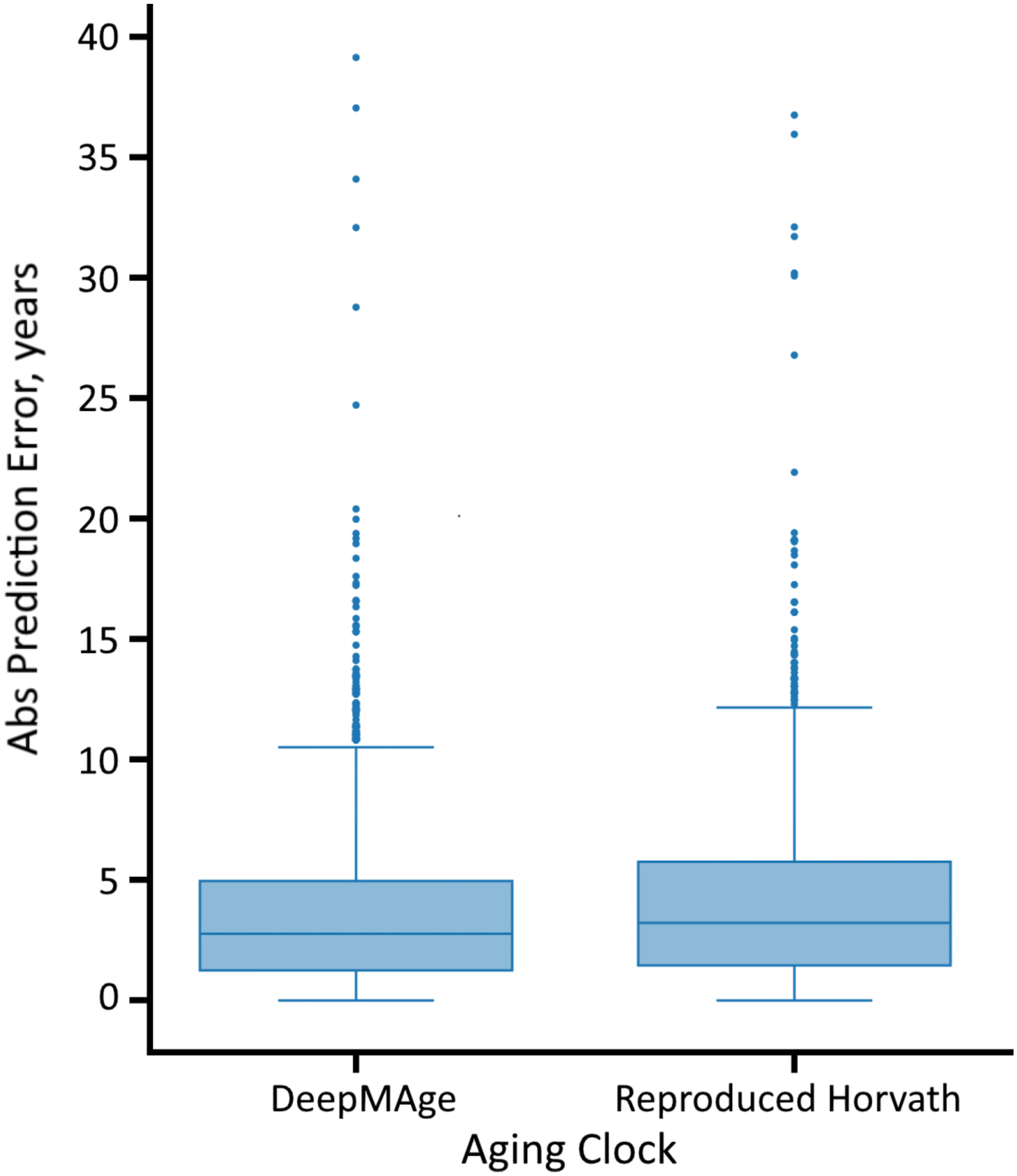

Supplementary Figure 6. Boxplots for absolute prediction errors in the DeepMAge and the *de novo* elastic net regressor reproduced according to Horvath's protocol. The MAE of DeepMAge (3.80 years) was significantly lower (*p-value* = 0.0001) than that of the elastic net (4.24 years). The total number of samples used was 1,293. The middle lines represent the medians; the box edges represent the quartiles; the whiskers reach out no farther than 1.5 times the interquartile distance.
Abs = Absolute; MAE =Mean Absolute Error

© 2020. Galkin G et al. Published online at http://www.aginganddisease.org/EN/10.14336/AD.2020.1202

SUPPLEMENTARY DATA

Supplementary Table 1. Per-study report containing DeepMAge accuracy (MedAE in years), DeepMAge cohort, male ratio, and age range, as well as the baseline accuracy (median age assignment).

Study	Cohort	N, samples	Male ratio, %	Age range, years	MedAE, years	Baseline, years	Platform
GSE81961	train	40	0.0	21-43	2.62	3.65	450k
GSE52588	train	58	12.0	9-83	2.72	14.0	450k
GSE52588	case_train	29	62.0	10-43	2.82	8.0	450k
GSE97362	train	83	67.0	3-19	1.4	3.0	450k
GSE97362	case_train	150	61.0	0-52	3.47	5.5	450k
GSE41037	train	720	62.0	16-88	2.29	10.0	27k
GSE30870	train	39	0.0	0-103	2.96	14.0	450k
GSE61496	verification	310	53.0	30-74	2.14	16.5	450k
GSE98876	verification	71	100.0	26-69	2.54	6.0	450k
GSE37008	verification	99	37.0	24-45	3.74	4.0	27k
GSE128235	train	536	43.0	18-87	1.99	9.0	450k
GSE87640	case_verification	156	65.0	18-63	3.97	8.8	450k
GSE87640	verification	84	62.0	20-58	2.52	5.05	450k
GSE87582	case_verification	20	90.0	50-71	4.38	2.81	450k
GSE87582	verification	1	100.0	60-60	9.59	0.0	450k
GSE19711	train	272	0.0	52-78	4.25	6.0	27k
GSE19711	case_train	264	0.0	49-91	3.7	8.0	27k
GSE34639	verification	48	33.0	0-1	1.92	0.5	450k
GSE79329	verification	34	100.0	43-70	2.63	8.7	450k
GSE67530	train	105	53.0	22-93	4.43	12.0	450k
GSE67530	case_train	39	59.0	22-91	3.43	10.0	450k
GSE105123	verification	107	58	19-23	2.06	1.0	450k
GSE99624	case_verification	32	12.0	50-87	3.92	7.5	450k
GSE99624	verification	16	38.0	49-82	2.72	2.5	450k
GSE125105	train	688	45.0	17-87	2.1	11.0	450k
GSE102177	case_verification	18	61.0	4-10	1.84	0.53	450k
GSE102177	verification	18	56	4-14	1.87	2.0	450k
GSE20067	case_verification	195	49.0	24-74	4.99	6.0	27k
GSE27044	train	889	100.0	3-26	1.08	3.0	27k
GSE103911	verification	65	71.0	27-77	6.96	8.0	450k
GSE53740	train	197	32.0	37-93	2.95	7.0	450k
GSE53740	case_train	186	35.0	34-91	3.62	4.5	450k
GSE59065	verification	295	48.0	22-84	4.35	11.0	450k
GSE112696	case_verification	6	67.0	18-29	5.51	3.0	450k
GSE112696	verification	6	67.0	22-27	3.75	0.5	450k
GSE77696	train	117	88.0	27-76	4.24	5.0	450k
GSE77696	case_train	261	96.0	25-75	4.25	6.0	450k
GSE58119	train	282	0.0	50-75	3.89	5.0	27k
GSE106648	train	139	25.0	20-65	2.48	7.0	450k
GSE106648	case_train	140	30.0	16-66	1.74	9.0	450k
GSE77445	train	85	51.0	18-69	2.7	4.0	450k
GSE84624	train	24	50.0	0-5	1.32	0.42	450k
GSE84624	case_train	24	54.0	0-7	1.27	0.9	450k
GSE107737	case_verification	12	100.0	18-27	2.46	2.0	450k
GSE107737	verification	12	100.0	18-29	3.03	3.5	450k
GSE40279	train	656	48.0	19-101	4.25	11.0	450k
GSE107459	verification	127	0.0	18-35	1.63	2.72	450k

© 2020. Galkin G et al. Published online at http://www.aginganddisease.org/EN/10.14336/AD.2020.1202

SUPPLEMENTARY DATA

Supplementary Table 2. 1,000 CpG sites comprising DeepMAge, ranked by feature importance (higher importance features come first).

Rank	CpG site	Importance	Rank	CpG site	Importance	Rank	CpG site	Importance	Rank	CpG site	Importance
1	cg01580888	0.000149323	31	cg15319457	7.45E-05	61	cg08209133	6.14E-05	91	cg00462994	5.47E-05
2	cg21801378	0.000143596	32	cg11668844	7.41E-05	62	cg18182399	6.13E-05	92	cg11377136	5.45E-05
3	cg00343092	0.000143059	33	cg07850604	7.32E-05	63	cg07388493	6.11E-05	93	cg25809905	5.44E-05
4	cg26394940	0.00012294	34	cg27015931	7.21E-05	64	cg17729667	6.11E-05	94	cg25564800	5.43E-05
5	cg12024906	0.000120211	35	cg19046959	7.18E-05	65	cg26372517	6.08E-05	95	cg05436231	5.42E-05
6	cg22736354	0.000119079	36	cg08668790	7.05E-05	66	cg19885761	6.05E-05	96	cg00987379	5.42E-05
7	cg18815943	0.000107798	37	cg01511567	6.88E-05	67	cg26842024	5.99E-05	97	cg01820374	5.40E-05
8	cg13269407	0.000107461	38	cg00503840	6.81E-05	68	cg23303074	5.95E-05	98	cg12238343	5.39E-05
9	cg06493994	0.000106796	39	cg20143092	6.77E-05	69	cg24826867	5.95E-05	99	cg13975369	5.36E-05
10	cg10523019	9.53E-05	40	cg12373771	6.76E-05	70	cg10362475	5.94E-05	100	cg23887396	5.29E-05
11	cg27491887	9.13E-05	41	cg15957394	6.76E-05	71	cg11299964	5.92E-05	101	cg04662594	5.29E-05
12	cg17861230	9.11E-05	42	cg18902090	6.72E-05	72	cg22947000	5.91E-05	102	cg03330058	5.27E-05
13	cg04836038	8.82E-05	43	cg15013019	6.70E-05	73	cg06268694	5.90E-05	103	cg00930873	5.26E-05
14	cg09809672	8.76E-05	44	cg03623878	6.66E-05	74	cg16785344	5.88E-05	104	cg08468689	5.26E-05
15	cg02479575	8.66E-05	45	cg18267374	6.64E-05	75	cg19724470	5.81E-05	105	cg06836772	5.25E-05
16	cg21296230	8.63E-05	46	cg02397514	6.57E-05	76	cg07158339	5.76E-05	106	cg08694544	5.24E-05
17	cg19722847	8.50E-05	47	cg15804973	6.53E-05	77	cg26614073	5.75E-05	107	cg13931228	5.23E-05
18	cg27320127	8.47E-05	48	cg16744741	6.53E-05	78	cg26845300	5.74E-05	108	cg01530101	5.22E-05
19	cg05675373	8.29E-05	49	cg25148589	6.44E-05	79	cg05822532	5.70E-05	109	cg03975694	5.22E-05
20	cg18008766	8.26E-05	50	cg18055007	6.42E-05	80	cg21790626	5.65E-05	110	cg08317263	5.22E-05
21	cg24127874	8.09E-05	51	cg00059225	6.40E-05	81	cg18660898	5.65E-05	111	cg12339802	5.19E-05
22	cg13663218	7.87E-05	52	cg24081819	6.35E-05	82	cg02310296	5.63E-05	112	cg12946225	5.19E-05
23	cg19560758	7.87E-05	53	cg27544190	6.30E-05	83	cg21368354	5.62E-05	113	cg04431054	5.17E-05
24	cg11126134	7.77E-05	54	cg18236477	6.27E-05	84	cg16313343	5.58E-05	114	cg05135156	5.13E-05
25	cg22407458	7.75E-05	55	cg06291867	6.26E-05	85	cg16273597	5.56E-05	115	cg14918082	5.08E-05
26	cg18691434	7.74E-05	56	cg07211259	6.25E-05	86	cg04123409	5.56E-05	116	cg08965235	5.05E-05
27	cg19761273	7.66E-05	57	cg13494498	6.23E-05	87	cg08090640	5.49E-05	117	cg10947146	5.04E-05
28	cg24891133	7.53E-05	58	cg10189695	6.21E-05	88	cg18440048	5.48E-05	118	cg13460409	5.04E-05
29	cg04528819	7.47E-05	59	cg12422450	6.17E-05	89	cg20300246	5.47E-05	119	cg06156376	5.04E-05
30	cg17285325	7.47E-05	60	cg24170090	6.14E-05	90	cg20761322	5.47E-05	120	cg01899253	5.03E-05

Rank	CpG site	Importance	Rank	CpG site	Importance	Rank	CpG site	Importance	Rank	CpG site	Importance
121	cg08695830	5.01E-05	151	cg13921352	4.67E-05	181	cg02154074	4.40E-05	211	cg07313155	4.21E-05
122	cg04872689	5.00E-05	152	cg21870884	4.66E-05	182	cg21448423	4.40E-05	212	cg08186362	4.20E-05
123	cg10734665	4.98E-05	153	cg13302154	4.65E-05	183	cg09949775	4.39E-05	213	cg09626984	4.20E-05

© 2020. Galkin G et al. Published online at http://www.aginganddisease.org/EN/10.14336/AD.2020.1202

SUPPLEMENTARY DATA

124	cg15361590	4.98E-05	154	cg07895149	4.64E-05	184	cg02840794	4.39E-05	214	cg25141674	4.17E-05
125	cg15201877	4.97E-05	155	cg07715201	4.64E-05	185	cg21581873	4.39E-05	215	cg16933388	4.17E-05
126	cg18992688	4.97E-05	156	cg01295203	4.64E-05	186	cg17410236	4.38E-05	216	cg02096633	4.17E-05
127	cg17051321	4.95E-05	157	cg16670497	4.62E-05	187	cg25332298	4.37E-05	217	cg23843812	4.16E-05
128	cg03664992	4.91E-05	158	cg16786458	4.62E-05	188	cg00194146	4.37E-05	218	cg17832674	4.15E-05
129	cg00565688	4.89E-05	159	cg13129046	4.61E-05	189	cg26599006	4.36E-05	219	cg20295671	4.15E-05
130	cg04425624	4.87E-05	160	cg23290344	4.61E-05	190	cg27316956	4.36E-05	220	cg19423311	4.14E-05
131	cg21256649	4.87E-05	161	cg04474832	4.59E-05	191	cg05266781	4.36E-05	221	cg23124451	4.13E-05
132	cg03734874	4.87E-05	162	cg22392276	4.58E-05	192	cg19357849	4.32E-05	222	cg24989962	4.13E-05
133	cg02844545	4.87E-05	163	cg15379633	4.58E-05	193	cg24871743	4.32E-05	223	cg22809047	4.13E-05
134	cg20125091	4.86E-05	164	cg19211800	4.57E-05	194	cg23178308	4.31E-05	224	cg04586023	4.13E-05
135	cg16516400	4.84E-05	165	cg20692569	4.56E-05	195	cg21700166	4.31E-05	225	cg10741760	4.13E-05
136	cg07408456	4.83E-05	166	cg22919728	4.54E-05	196	cg16168311	4.30E-05	226	cg11065385	4.12E-05
137	cg12145907	4.83E-05	167	cg26369667	4.51E-05	197	cg17133388	4.30E-05	227	cg03996822	4.11E-05
138	cg14754581	4.82E-05	168	cg27210390	4.51E-05	198	cg25499099	4.29E-05	228	cg22730004	4.11E-05
139	cg06263495	4.81E-05	169	cg09381003	4.51E-05	199	cg18693704	4.28E-05	229	cg03336167	4.10E-05
140	cg12402251	4.81E-05	170	cg02164046	4.51E-05	200	cg06458239	4.28E-05	230	cg07703401	4.08E-05
141	cg09643544	4.80E-05	171	cg25229172	4.50E-05	201	cg06738602	4.27E-05	231	cg17339202	4.08E-05
142	cg26005082	4.80E-05	172	cg13836627	4.50E-05	202	cg01777397	4.27E-05	232	cg17497271	4.07E-05
143	cg16731240	4.77E-05	173	cg12620499	4.49E-05	203	cg03688818	4.26E-05	233	cg01405761	4.05E-05
144	cg25763788	4.75E-05	174	cg13573276	4.49E-05	204	cg06204948	4.24E-05	234	cg08900043	4.05E-05
145	cg14166009	4.75E-05	175	cg17940013	4.48E-05	205	cg25985778	4.24E-05	235	cg08529529	4.05E-05
146	cg02151301	4.74E-05	176	cg24199834	4.47E-05	206	cg02228185	4.24E-05	236	cg17471102	4.04E-05
147	cg26610808	4.71E-05	177	cg04270799	4.45E-05	207	cg16363586	4.22E-05	237	cg22892904	4.03E-05
148	cg10316635	4.71E-05	178	cg08888956	4.44E-05	208	cg26151675	4.22E-05	238	cg24968336	3.99E-05
149	cg22171829	4.70E-05	179	cg23710218	4.43E-05	209	cg23967169	4.22E-05	239	cg00236832	3.98E-05
150	cg17199483	4.69E-05	180	cg11896923	4.42E-05	210	cg24921089	4.22E-05	240	cg15898840	3.97E-05

**Rank**	**CpG site**	**Importance**	**Rank**	**CpG site**	**Importance**	**Rank**	**CpG site**	**Importance**	**Rank**	**CpG site**	**Importance**
241	cg24471894	3.96E-05	271	cg23828595	3.75E-05	301	cg21697134	3.58E-05	331	cg15743985	3.44E-05
242	cg03991512	3.96E-05	272	cg14592406	3.75E-05	302	cg04601137	3.57E-05	332	cg23854009	3.43E-05
243	cg22285621	3.94E-05	273	cg10822172	3.75E-05	303	cg24169822	3.57E-05	333	cg19008809	3.43E-05
244	cg23843505	3.93E-05	274	cg05064673	3.75E-05	304	cg27360098	3.56E-05	334	cg23668631	3.42E-05
245	cg11378686	3.92E-05	275	cg09554443	3.74E-05	305	cg01968178	3.55E-05	335	cg27153400	3.42E-05
246	cg19515518	3.92E-05	276	cg05369142	3.74E-05	306	cg02217159	3.55E-05	336	cg11946503	3.42E-05
247	cg23211240	3.92E-05	277	cg17274064	3.73E-05	307	cg13697378	3.55E-05	337	cg00081975	3.41E-05
248	cg23189044	3.92E-05	278	cg23517605	3.73E-05	308	cg25044651	3.54E-05	338	cg14175438	3.41E-05
249	cg09118625	3.91E-05	279	cg21992250	3.73E-05	309	cg16319578	3.54E-05	339	cg17688525	3.41E-05

© 2020. Galkin G et al. Published online at http://www.aginganddisease.org/EN/10.14336/AD.2020.1202

SUPPLEMENTARY DATA

250	cg04765422	3.91E-05	280	cg20974196	3.72E-05	310	cg09067967	3.54E-05	340	cg27553955	3.41E-05
251	cg26911787	3.91E-05	281	cg11120551	3.72E-05	311	cg12688670	3.54E-05	341	cg05767404	3.41E-05
252	cg11536940	3.90E-05	282	cg11919694	3.69E-05	312	cg03891319	3.53E-05	342	cg27016307	3.40E-05
253	cg25822709	3.90E-05	283	cg14319409	3.68E-05	313	cg18919097	3.53E-05	343	cg12782180	3.40E-05
254	cg14456683	3.89E-05	284	cg16620032	3.67E-05	314	cg09736162	3.53E-05	344	cg16465939	3.40E-05
255	cg15297650	3.88E-05	285	cg19789466	3.67E-05	315	cg14261309	3.53E-05	345	cg03224418	3.40E-05
256	cg23587449	3.88E-05	286	cg25459323	3.66E-05	316	cg26500816	3.52E-05	346	cg26963271	3.39E-05
257	cg05881135	3.86E-05	287	cg19356189	3.66E-05	317	cg25538571	3.52E-05	347	cg01407797	3.39E-05
258	cg01283289	3.86E-05	288	cg03544320	3.66E-05	318	cg09915099	3.51E-05	348	cg08822227	3.38E-05
259	cg10549973	3.83E-05	289	cg02364642	3.64E-05	319	cg23428445	3.50E-05	349	cg06320982	3.38E-05
260	cg25256723	3.82E-05	290	cg18755783	3.64E-05	320	cg16614500	3.48E-05	350	cg05535113	3.38E-05
261	cg03929796	3.81E-05	291	cg03030757	3.63E-05	321	cg19235307	3.47E-05	351	cg21096915	3.36E-05
262	cg13854874	3.80E-05	292	cg09462576	3.63E-05	322	cg08876932	3.46E-05	352	cg03909500	3.36E-05
263	cg14332079	3.78E-05	293	cg05379350	3.63E-05	323	cg10235817	3.46E-05	353	cg06147863	3.36E-05
264	cg01946401	3.78E-05	294	cg05158615	3.60E-05	324	cg01459453	3.46E-05	354	cg20240860	3.36E-05
265	cg01294695	3.78E-05	295	cg24860534	3.60E-05	325	cg19055231	3.46E-05	355	cg03943081	3.35E-05
266	cg07123069	3.77E-05	296	cg16682903	3.60E-05	326	cg24851490	3.45E-05	356	cg01154193	3.35E-05
267	cg18573383	3.77E-05	297	cg02489552	3.60E-05	327	cg15839448	3.45E-05	357	cg06361108	3.34E-05
268	cg01400401	3.77E-05	298	cg22527345	3.59E-05	328	cg00489401	3.45E-05	358	cg24012925	3.33E-05
269	cg00047050	3.76E-05	299	cg20008332	3.59E-05	329	cg04062391	3.45E-05	359	cg17791651	3.33E-05
270	cg23506842	3.75E-05	300	cg05442902	3.59E-05	330	cg22396353	3.45E-05	360	cg20979799	3.32E-05

**Rank**	**CpG site**	**Importance**	**Rank**	**CpG site**	**Importance**	**Rank**	**CpG site**	**Importance**	**Rank**	**CpG site**	**Importance**
361	cg12365667	3.32E-05	391	cg17207590	3.22E-05	421	cg27389185	3.13E-05	451	cg26297688	3.07E-05
362	cg17031727	3.32E-05	392	cg09072120	3.21E-05	422	cg00308665	3.13E-05	452	cg25736482	3.06E-05
363	cg18059933	3.32E-05	393	cg10927536	3.21E-05	423	cg10150813	3.13E-05	453	cg00911351	3.06E-05
364	cg25947945	3.31E-05	394	cg20264732	3.20E-05	424	cg06433658	3.13E-05	454	cg05010623	3.05E-05
365	cg25766046	3.31E-05	395	cg25282410	3.20E-05	425	cg12758687	3.13E-05	455	cg11808757	3.05E-05
366	cg09427311	3.31E-05	396	cg14859417	3.20E-05	426	cg09262269	3.12E-05	456	cg05570980	3.05E-05
367	cg26304237	3.31E-05	397	cg12774845	3.20E-05	427	cg13885201	3.12E-05	457	cg00426498	3.04E-05
368	cg22747092	3.30E-05	398	cg12741420	3.20E-05	428	cg18787975	3.12E-05	458	cg05890019	3.04E-05
369	cg19713196	3.30E-05	399	cg04424621	3.19E-05	429	cg20973210	3.12E-05	459	cg14967066	3.04E-05
370	cg19402885	3.30E-05	400	cg17878972	3.19E-05	430	cg06971096	3.11E-05	460	cg18074297	3.04E-05
371	cg19310430	3.29E-05	401	cg21530890	3.19E-05	431	cg15563382	3.11E-05	461	cg19395441	3.04E-05
372	cg24653181	3.29E-05	402	cg25166896	3.19E-05	432	cg10281002	3.11E-05	462	cg03565323	3.03E-05
373	cg19945840	3.29E-05	403	cg16543027	3.18E-05	433	cg15982419	3.11E-05	463	cg17453778	3.03E-05
374	cg21870662	3.28E-05	404	cg21057046	3.18E-05	434	cg15928398	3.10E-05	464	cg24231716	3.03E-05
375	cg15903421	3.28E-05	405	cg09816471	3.18E-05	435	cg17992056	3.10E-05	465	cg05473871	3.03E-05

© 2020. Galkin G et al. Published online at http://www.aginganddisease.org/EN/10.14336/AD.2020.1202

SUPPLEMENTARY DATA

376	cg04289385	3.28E-05	406	cg10193817	3.18E-05	436	cg11981599	3.10E-05	466	cg22187630	3.02E-05
377	cg12870705	3.28E-05	407	cg25802093	3.17E-05	437	cg00168942	3.09E-05	467	cg05250458	3.02E-05
378	cg04329454	3.27E-05	408	cg01519742	3.16E-05	438	cg25375711	3.09E-05	468	cg07935568	3.02E-05
379	cg20158248	3.26E-05	409	cg12941369	3.16E-05	439	cg12532500	3.09E-05	469	cg02620013	3.02E-05
380	cg10319505	3.26E-05	410	cg25511429	3.16E-05	440	cg10044101	3.09E-05	470	cg21016177	3.02E-05
381	cg12078929	3.25E-05	411	cg09660171	3.15E-05	441	cg00201234	3.08E-05	471	cg03848555	3.01E-05
382	cg15377518	3.25E-05	412	cg22705225	3.15E-05	442	cg07139440	3.08E-05	472	cg18016365	3.01E-05
383	cg07099407	3.24E-05	413	cg15415507	3.15E-05	443	cg22909609	3.08E-05	473	cg21908259	3.01E-05
384	cg08570521	3.24E-05	414	cg03641225	3.15E-05	444	cg20449692	3.08E-05	474	cg24739326	3.01E-05
385	cg12261786	3.24E-05	415	cg14386691	3.15E-05	445	cg15473868	3.08E-05	475	cg18303397	3.01E-05
386	cg02789485	3.23E-05	416	cg08896945	3.14E-05	446	cg02197293	3.07E-05	476	cg10756887	3.00E-05
387	cg19759064	3.23E-05	417	cg25983380	3.14E-05	447	cg22449114	3.07E-05	477	cg17838026	3.00E-05
388	cg24384676	3.23E-05	418	cg22115808	3.14E-05	448	cg05228408	3.07E-05	478	cg13666340	3.00E-05
389	cg02564523	3.22E-05	419	cg18678185	3.13E-05	449	cg16924616	3.07E-05	479	cg10722799	3.00E-05
390	cg06810647	3.22E-05	420	cg11438428	3.13E-05	450	cg12259537	3.07E-05	480	cg01200177	3.00E-05
**Rank**	**CpG site**	**Importance**	**Rank**	**CpG site**	**Importance**	**Rank**	**CpG site**	**Importance**	**Rank**	**CpG site**	**Importance**
481	cg03852144	2.99E-05	511	cg15037004	2.94E-05	541	cg13500819	2.88E-05	571	cg16776350	2.82E-05
482	cg18511007	2.99E-05	512	cg23833896	2.94E-05	542	cg06824727	2.88E-05	572	cg23265096	2.82E-05
483	cg00202702	2.99E-05	513	cg10865119	2.94E-05	543	cg00563926	2.88E-05	573	cg00548268	2.81E-05
484	cg26824091	2.99E-05	514	cg14865868	2.94E-05	544	cg08655844	2.88E-05	574	cg12052765	2.81E-05
485	cg02848777	2.99E-05	515	cg10281478	2.93E-05	545	cg07903918	2.88E-05	575	cg25302419	2.81E-05
486	cg25054311	2.98E-05	516	cg25942450	2.93E-05	546	cg04460372	2.87E-05	576	cg18765542	2.81E-05
487	cg08022502	2.98E-05	517	cg22613010	2.93E-05	547	cg16483916	2.87E-05	577	cg21289015	2.81E-05
488	cg02085507	2.98E-05	518	cg22901840	2.93E-05	548	cg11279021	2.87E-05	578	cg20043466	2.80E-05
489	cg10682057	2.98E-05	519	cg20001829	2.93E-05	549	cg11189837	2.87E-05	579	cg02071305	2.80E-05
490	cg10084993	2.98E-05	520	cg25604883	2.93E-05	550	cg27601516	2.87E-05	580	cg01805282	2.80E-05
491	cg20994801	2.98E-05	521	cg12513481	2.92E-05	551	cg24056567	2.86E-05	581	cg07442479	2.80E-05
492	cg15156836	2.98E-05	522	cg13899108	2.92E-05	552	cg20279283	2.86E-05	582	cg17431739	2.80E-05
493	cg06269753	2.98E-05	523	cg05871136	2.92E-05	553	cg16063112	2.86E-05	583	cg24642523	2.80E-05
494	cg22680204	2.98E-05	524	cg05483509	2.92E-05	554	cg24986868	2.86E-05	584	cg10240853	2.80E-05
495	cg26036443	2.97E-05	525	cg16254309	2.91E-05	555	cg00431114	2.86E-05	585	cg09595479	2.79E-05
496	cg02828104	2.97E-05	526	cg27281093	2.91E-05	556	cg00563932	2.86E-05	586	cg23320649	2.79E-05
497	cg16270890	2.97E-05	527	cg12556134	2.91E-05	557	cg19706682	2.85E-05	587	cg08996521	2.79E-05
498	cg17324128	2.97E-05	528	cg20900524	2.91E-05	558	cg15747595	2.85E-05	588	cg15776355	2.79E-05
499	cg08303146	2.97E-05	529	cg11584690	2.91E-05	559	cg16352283	2.85E-05	589	cg20654468	2.79E-05
500	cg07195577	2.97E-05	530	cg03600687	2.91E-05	560	cg26131019	2.85E-05	590	cg09429111	2.79E-05
501	cg25713185	2.97E-05	531	cg19283196	2.91E-05	561	cg06638433	2.84E-05	591	cg23850212	2.79E-05

© 2020. Galkin G et al. Published online at http://www.aginganddisease.org/EN/10.14336/AD.2020.1202

502	cg14826456	2.96E-05	532	cg03883519	2.90E-05	562	cg00689340	2.84E-05	592	cg16240480	2.79E-05
503	cg27169020	2.95E-05	533	cg19594666	2.90E-05	563	cg27187881	2.84E-05	593	cg07185695	2.78E-05
504	cg07430605	2.95E-05	534	cg10515956	2.90E-05	564	cg11879514	2.83E-05	594	cg12073594	2.78E-05
505	cg09492887	2.95E-05	535	cg16362133	2.90E-05	565	cg13593287	2.83E-05	595	cg15201635	2.78E-05
506	cg05010058	2.95E-05	536	cg07737781	2.89E-05	566	cg06948294	2.83E-05	596	cg23762517	2.78E-05
507	cg10226744	2.95E-05	537	cg11314684	2.89E-05	567	cg03565081	2.83E-05	597	cg15352829	2.78E-05
508	cg02206259	2.95E-05	538	cg14377791	2.89E-05	568	cg06161930	2.82E-05	598	cg20346726	2.78E-05
509	cg17471928	2.94E-05	539	cg19355190	2.89E-05	569	cg11010122	2.82E-05	599	cg11738543	2.77E-05
510	cg20637307	2.94E-05	540	cg11747499	2.88E-05	570	cg24512400	2.82E-05	600	cg00208967	2.77E-05

Rank	CpG site	Importance	Rank	CpG site	Importance	Rank	CpG site	Importance	Rank	CpG site	Importance
601	cg03782453	2.77E-05	631	cg01654582	2.71E-05	661	cg09325711	2.64E-05	691	cg09563216	2.60E-05
602	cg19713460	2.77E-05	632	cg00340102	2.71E-05	662	cg23239396	2.64E-05	692	cg06144905	2.59E-05
603	cg05600717	2.77E-05	633	cg03826976	2.70E-05	663	cg14155397	2.64E-05	693	cg09706243	2.59E-05
604	cg04786857	2.76E-05	634	cg14870271	2.70E-05	664	cg17029151	2.64E-05	694	cg01919208	2.59E-05
605	cg02335441	2.76E-05	635	cg02654291	2.70E-05	665	cg13620770	2.64E-05	695	cg11428724	2.59E-05
606	cg16127845	2.76E-05	636	cg04036898	2.70E-05	666	cg15974053	2.64E-05	696	cg12928668	2.59E-05
607	cg22631938	2.76E-05	637	cg14992253	2.69E-05	667	cg01161216	2.64E-05	697	cg00090147	2.59E-05
608	cg21426387	2.76E-05	638	cg12613383	2.69E-05	668	cg08849574	2.64E-05	698	cg00630583	2.59E-05
609	cg22472290	2.76E-05	639	cg10917602	2.69E-05	669	cg00152644	2.63E-05	699	cg14958635	2.59E-05
610	cg09340639	2.76E-05	640	cg07652213	2.68E-05	670	cg17966619	2.63E-05	700	cg26083396	2.59E-05
611	cg08587864	2.76E-05	641	cg21820677	2.68E-05	671	cg26780333	2.63E-05	701	cg20080624	2.58E-05
612	cg19168338	2.76E-05	642	cg14681055	2.68E-05	672	cg20419410	2.63E-05	702	cg08370996	2.58E-05
613	cg25725843	2.75E-05	643	cg19635712	2.68E-05	673	cg20227766	2.63E-05	703	cg23430664	2.58E-05
614	cg20616414	2.75E-05	644	cg13726191	2.68E-05	674	cg24127989	2.63E-05	704	cg19889780	2.58E-05
615	cg06675478	2.75E-05	645	cg05164634	2.67E-05	675	cg23752923	2.63E-05	705	cg24200059	2.58E-05
616	cg20209009	2.75E-05	646	cg19155599	2.67E-05	676	cg15456206	2.63E-05	706	cg14100184	2.58E-05
617	cg04598121	2.75E-05	647	cg01269795	2.67E-05	677	cg24727203	2.62E-05	707	cg13047892	2.58E-05
618	cg00564163	2.75E-05	648	cg19764555	2.67E-05	678	cg04739570	2.62E-05	708	cg04457979	2.58E-05
619	cg20496643	2.75E-05	649	cg22236626	2.67E-05	679	cg05056120	2.61E-05	709	cg14056644	2.57E-05
620	cg01027739	2.74E-05	650	cg11260848	2.66E-05	680	cg17692403	2.61E-05	710	cg19669036	2.57E-05
621	cg02503850	2.74E-05	651	cg07621046	2.66E-05	681	cg17914753	2.60E-05	711	cg04597449	2.57E-05
622	cg12902039	2.74E-05	652	cg22719623	2.66E-05	682	cg27493997	2.60E-05	712	cg07979752	2.56E-05
623	cg06238491	2.73E-05	653	cg09083627	2.66E-05	683	cg02988947	2.60E-05	713	cg00685836	2.56E-05
624	cg24587268	2.73E-05	654	cg11833861	2.65E-05	684	cg02016419	2.60E-05	714	cg09079275	2.56E-05
625	cg04880063	2.73E-05	655	cg01580044	2.65E-05	685	cg10362591	2.60E-05	715	cg04726200	2.56E-05
626	cg26711820	2.72E-05	656	cg05546044	2.65E-05	686	cg22521310	2.60E-05	716	cg26673195	2.56E-05
627	cg25655096	2.72E-05	657	cg13745346	2.64E-05	687	cg06051311	2.60E-05	717	cg12069309	2.56E-05

© 2020. Galkin G et al. Published online at http://www.aginganddisease.org/EN/10.14336/AD.2020.1202

SUPPLEMENTARY DATA

628	cg09601629	2.72E-05	658	cg20831708	2.64E-05	688	cg02515725	2.60E-05	718	cg23283875	2.56E-05
629	cg19233923	2.72E-05	659	cg08555657	2.64E-05	689	cg22321558	2.60E-05	719	cg02994956	2.55E-05
630	cg25629694	2.72E-05	660	cg19573166	2.64E-05	690	cg07588779	2.60E-05	720	cg21480743	2.55E-05
**Rank**	**CpG site**	**Importance**	**Rank**	**CpG site**	**Importance**	**Rank**	**CpG site**	**Importance**	**Rank**	**CpG site**	**Importance**
721	cg11896271	2.55E-05	751	cg22971191	2.52E-05	781	cg02776251	2.48E-05	811	cg16761581	2.45E-05
722	cg02181506	2.55E-05	752	cg22436229	2.52E-05	782	cg10104451	2.48E-05	812	cg07314414	2.45E-05
723	cg00497251	2.55E-05	753	cg01600189	2.52E-05	783	cg15945417	2.48E-05	813	cg03945800	2.45E-05
724	cg21808053	2.55E-05	754	cg00651216	2.51E-05	784	cg17589341	2.48E-05	814	cg26512148	2.44E-05
725	cg15316334	2.55E-05	755	cg24076884	2.51E-05	785	cg06253072	2.47E-05	815	cg23047271	2.44E-05
726	cg16408970	2.55E-05	756	cg12955583	2.51E-05	786	cg24173049	2.47E-05	816	cg02774439	2.44E-05
727	cg15261665	2.54E-05	757	cg03760483	2.51E-05	787	cg02062650	2.47E-05	817	cg06621358	2.43E-05
728	cg05373457	2.54E-05	758	cg06392241	2.51E-05	788	cg03138091	2.47E-05	818	cg05898524	2.43E-05
729	cg25483003	2.54E-05	759	cg14913925	2.51E-05	789	cg07973967	2.47E-05	819	cg01346152	2.43E-05
730	cg01114088	2.54E-05	760	cg24429836	2.51E-05	790	cg15853125	2.47E-05	820	cg20557202	2.43E-05
731	cg19037167	2.54E-05	761	cg23758485	2.50E-05	791	cg06236061	2.47E-05	821	cg17943999	2.43E-05
732	cg02255609	2.54E-05	762	cg07846167	2.50E-05	792	cg18555440	2.47E-05	822	cg00398048	2.43E-05
733	cg11648289	2.54E-05	763	cg22101147	2.50E-05	793	cg00282347	2.46E-05	823	cg08441806	2.43E-05
734	cg09582042	2.54E-05	764	cg19728223	2.50E-05	794	cg11223252	2.46E-05	824	cg12600197	2.43E-05
735	cg21353232	2.54E-05	765	cg07469792	2.50E-05	795	cg01017147	2.46E-05	825	cg00187380	2.43E-05
736	cg26018901	2.53E-05	766	cg13311440	2.49E-05	796	cg06117855	2.46E-05	826	cg16998353	2.43E-05
737	cg21818252	2.53E-05	767	cg07482936	2.49E-05	797	cg24768561	2.46E-05	827	cg26509022	2.43E-05
738	cg14348532	2.53E-05	768	cg24646414	2.49E-05	798	cg02276665	2.46E-05	828	cg04466273	2.43E-05
739	cg13565157	2.53E-05	769	cg26928682	2.49E-05	799	cg21509097	2.46E-05	829	cg14093936	2.43E-05
740	cg02764611	2.53E-05	770	cg16386080	2.49E-05	800	cg18972811	2.46E-05	830	cg00472814	2.43E-05
741	cg05488632	2.53E-05	771	cg03547797	2.49E-05	801	cg00576250	2.46E-05	831	cg27236973	2.43E-05
742	cg21120249	2.53E-05	772	cg06630241	2.49E-05	802	cg09155852	2.46E-05	832	cg23786576	2.42E-05
743	cg08569678	2.53E-05	773	cg08097882	2.48E-05	803	cg02254649	2.46E-05	833	cg12457773	2.42E-05
744	cg26624134	2.53E-05	774	cg08646988	2.48E-05	804	cg07495664	2.46E-05	834	cg09863772	2.42E-05
745	cg13163729	2.52E-05	775	cg17830308	2.48E-05	805	cg24450312	2.46E-05	835	cg26209676	2.42E-05
746	cg07753644	2.52E-05	776	cg20028470	2.48E-05	806	cg15271616	2.46E-05	836	cg10194829	2.42E-05
747	cg06154570	2.52E-05	777	cg15720535	2.48E-05	807	cg26968812	2.45E-05	837	cg21073927	2.42E-05
748	cg05294243	2.52E-05	778	cg21604042	2.48E-05	808	cg05786809	2.45E-05	838	cg27626102	2.42E-05
749	cg22580512	2.52E-05	779	cg24801210	2.48E-05	809	cg11469321	2.45E-05	839	cg21402071	2.42E-05
750	cg00107187	2.52E-05	780	cg14973995	2.48E-05	810	cg07558455	2.45E-05	840	cg17165284	2.42E-05
**Rank**	**CpG site**	**Importance**	**Rank**	**CpG site**	**Importance**	**Rank**	**CpG site**	**Importance**	**Rank**	**CpG site**	**Importance**
841	cg16332577	2.41E-05	871	cg23748737	2.39E-05	901	cg01655355	2.36E-05	931	cg04409945	2.33E-05
842	cg14540297	2.41E-05	872	cg19464016	2.39E-05	902	cg03775422	2.36E-05	932	cg08654655	2.32E-05

© 2020. Galkin G et al. Published online at http://www.aginganddisease.org/EN/10.14336/AD.2020.1202

SUPPLEMENTARY DATA

843	cg17421623	2.41E-05	873	cg23002907	2.39E-05	903	cg01441777	2.36E-05	933	cg21176048	2.32E-05
844	cg21974766	2.41E-05	874	cg16427670	2.39E-05	904	cg20723355	2.36E-05	934	cg12331389	2.32E-05
845	cg02196655	2.41E-05	875	cg06385087	2.39E-05	905	cg01791232	2.36E-05	935	cg27631256	2.32E-05
846	cg26202340	2.41E-05	876	cg10648908	2.38E-05	906	cg22215728	2.36E-05	936	cg18081258	2.32E-05
847	cg26374101	2.41E-05	877	cg18464137	2.38E-05	907	cg24207176	2.36E-05	937	cg07991621	2.32E-05
848	cg11480873	2.41E-05	878	cg06288351	2.38E-05	908	cg13262687	2.36E-05	938	cg22799850	2.32E-05
849	cg07349094	2.41E-05	879	cg04114315	2.38E-05	909	cg12564453	2.36E-05	939	cg08097755	2.32E-05
850	cg15364618	2.41E-05	880	cg04032226	2.38E-05	910	cg11296937	2.36E-05	940	cg24874111	2.31E-05
851	cg25050026	2.41E-05	881	cg23146358	2.38E-05	911	cg14972143	2.35E-05	941	cg08587542	2.31E-05
852	cg05724065	2.40E-05	882	cg11108890	2.38E-05	912	cg11041457	2.35E-05	942	cg25713309	2.31E-05
853	cg10175795	2.40E-05	883	cg11158729	2.38E-05	913	cg24107665	2.35E-05	943	cg01353448	2.31E-05
854	cg17338403	2.40E-05	884	cg10080004	2.38E-05	914	cg00653387	2.35E-05	944	cg20506783	2.31E-05
855	cg05001145	2.40E-05	885	cg10052840	2.38E-05	915	cg05073035	2.35E-05	945	cg04588079	2.31E-05
856	cg17169998	2.40E-05	886	cg00399483	2.38E-05	916	cg16404106	2.35E-05	946	cg26898166	2.31E-05
857	cg13234863	2.40E-05	887	cg05769161	2.38E-05	917	cg16954341	2.35E-05	947	cg05157725	2.31E-05
858	cg05868799	2.40E-05	888	cg08572611	2.38E-05	918	cg21926138	2.35E-05	948	cg08197122	2.31E-05
859	cg21949781	2.40E-05	889	cg26270746	2.37E-05	919	cg02755525	2.34E-05	949	cg00565075	2.31E-05
860	cg17252960	2.40E-05	890	cg06911084	2.37E-05	920	cg26093148	2.34E-05	950	cg10331779	2.30E-05
861	cg13548361	2.40E-05	891	cg18678763	2.37E-05	921	cg03889226	2.34E-05	951	cg02782630	2.30E-05
862	cg15003434	2.40E-05	892	cg10989517	2.37E-05	922	cg16984944	2.34E-05	952	cg20083676	2.30E-05
863	cg10287137	2.40E-05	893	cg16721845	2.37E-05	923	cg14913610	2.34E-05	953	cg12478185	2.30E-05
864	cg08724517	2.40E-05	894	cg07845392	2.37E-05	924	cg10893437	2.34E-05	954	cg05824484	2.30E-05
865	cg27376271	2.40E-05	895	cg13438834	2.37E-05	925	cg13526007	2.34E-05	955	cg24641352	2.30E-05
866	cg03379131	2.40E-05	896	cg16284292	2.36E-05	926	cg16718678	2.33E-05	956	cg08162780	2.30E-05
867	cg26261431	2.39E-05	897	cg04887278	2.36E-05	927	cg19596204	2.33E-05	957	cg02260587	2.30E-05
868	cg21547708	2.39E-05	898	cg13904493	2.36E-05	928	cg06885782	2.33E-05	958	cg24649713	2.30E-05
869	cg11368643	2.39E-05	899	cg05924583	2.36E-05	929	cg05507459	2.33E-05	959	cg20051033	2.30E-05
870	cg16474696	2.39E-05	900	cg24125648	2.36E-05	930	cg19192120	2.33E-05	960	cg05697231	2.30E-05

**Rank**	**CpG site**	**Importance**	**Rank**	**CpG site**	**Importance**
961	cg21092687	2.30E-05	991	cg13818573	2.26E-05
962	cg14244577	2.30E-05	992	cg26581729	2.26E-05
963	cg14329157	2.30E-05	993	cg10521852	2.26E-05
964	cg18809289	2.29E-05	994	cg11386746	2.26E-05
965	cg13150977	2.29E-05	995	cg13806135	2.26E-05
966	cg10986043	2.29E-05	996	cg21053529	2.26E-05
967	cg21152671	2.29E-05	997	cg00650762	2.25E-05
968	cg26984624	2.29E-05	998	cg22183706	2.25E-05
969	cg24101578	2.29E-05	999	cg20537629	2.25E-05
970	cg20716064	2.29E-05	1000	cg08331960	2.25E-05

© 2020. Galkin G et al. Published online at http://www.aginganddisease.org/EN/10.14336/AD.2020.1202

971	cg02994974	2.29E-05
972	cg17655614	2.29E-05
973	cg22799321	2.28E-05
974	cg16413777	2.28E-05
975	cg17775235	2.28E-05
976	cg21972382	2.28E-05
977	cg10064162	2.28E-05
978	cg08858521	2.28E-05
979	cg24596472	2.28E-05
980	cg16774604	2.28E-05
981	cg10106284	2.28E-05
982	cg18993334	2.27E-05
983	cg16519321	2.27E-05
984	cg00582628	2.27E-05
985	cg05194726	2.27E-05
986	cg24715735	2.27E-05
987	cg04587910	2.27E-05
988	cg17241310	2.27E-05
989	cg14380517	2.27E-05
990	cg23771661	2.27E-05

Supplementary Table 3. Gene enrichment report obtained with Gene Ontology.

GO biological process complete	Homo sapiens - REFLIST (20851)	# (out of 849 uploaded)	# (expected)	over/under	Fold Enrichment	raw P-value	FDR
system development (GO:0048731)	4460	302	181.6	+	1.66	2.52E-20	4.02E-16
neurogenesis (GO:0022008)	1669	152	67.96	+	2.24	1.41E-19	1.12E-15
multicellular organism development (GO:0007275)	5080	328	206.84	+	1.59	2.51E-19	1.33E-15
generation of neurons (GO:0048699)	1565	145	63.72	+	2.28	2.60E-19	1.04E-15
anatomical structure development (GO:0048856)	5469	346	222.68	+	1.55	2.85E-19	9.09E-16
nervous system development (GO:0007399)	2395	191	97.52	+	1.96	9.46E-19	2.52E-15
regulation of multicellular organismal process (GO:0051239)	3211	233	130.74	+	1.78	2.50E-18	5.70E-15
developmental process (GO:0032502)	5918	363	240.97	+	1.51	2.83E-18	5.64E-15
biological regulation (GO:0065007)	12525	633	509.99	+	1.24	3.51E-18	6.22E-15

© 2020. Galkin G et al. Published online at http://www.aginganddisease.org/EN/10.14336/AD.2020.1202

neuron differentiation (GO:0030182)	1021	107	41.57	+	2.57	1.10E-17	1.76E-14
multicellular organismal process (GO:0032501)	7037	410	286.53	+	1.43	1.33E-17	1.94E-14
cellular process (GO:0009987)	15458	732	629.41	+	1.16	4.76E-17	6.33E-14
Unclassified (UNCLASSIFIED)	3041	46	123.82	-	0.37	8.99E-17	1.10E-13
biological_process (GO:0008150)	17810	803	725.18	+	1.11	8.99E-17	1.02E-13
animal organ development (GO:0048513)	3225	227	131.31	+	1.73	2.04E-16	2.17E-13
cell differentiation (GO:0030154)	3744	251	152.45	+	1.65	7.31E-16	7.30E-13
anatomical structure morphogenesis (GO:0009653)	2181	170	88.8	+	1.91	1.23E-15	1.16E-12
cellular developmental process (GO:0048869)	3798	252	154.64	+	1.63	2.17E-15	1.92E-12
regulation of localization (GO:0032879)	2838	202	115.56	+	1.75	6.47E-15	5.44E-12
regulation of biological process (GO:0050789)	11825	592	481.48	+	1.23	2.70E-14	2.15E-11
regulation of biological quality (GO:0065008)	4134	264	168.33	+	1.57	3.14E-14	2.38E-11
regulation of developmental process (GO:0050793)	2651	188	107.94	+	1.74	1.39E-13	1.01E-10
regulation of cellular process (GO:0050794)	11288	567	459.62	+	1.23	2.60E-13	1.80E-10
animal organ morphogenesis (GO:0009887)	973	93	39.62	+	2.35	2.99E-13	1.99E-10
signaling (GO:0023052)	5504	323	224.11	+	1.44	4.78E-13	3.05E-10
regulation of multicellular organismal development (GO:2000026)	2082	155	84.77	+	1.83	1.18E-12	7.23E-10
cell communication (GO:0007154)	5610	324	228.43	+	1.42	2.83E-12	1.67E-09
cellular response to stimulus (GO:0051716)	6801	374	276.92	+	1.35	1.05E-11	5.99E-09
regulation of cell differentiation (GO:0045595)	1857	139	75.61	+	1.84	1.55E-11	8.52E-09
response to stimulus (GO:0050896)	8572	449	349.03	+	1.29	1.57E-11	8.35E-09
regulation of cell development (GO:0060284)	966	88	39.33	+	2.24	1.62E-11	8.37E-09
cellular response to organic substance (GO:0071310)	2400	167	97.72	+	1.71	1.79E-11	8.94E-09
response to endogenous stimulus (GO:0009719)	1512	119	61.56	+	1.93	2.74E-11	1.32E-08
behavior (GO:0007610)	600	64	24.43	+	2.62	3.18E-11	1.49E-08
regulation of neurogenesis (GO:0050767)	840	79	34.2	+	2.31	4.67E-11	2.13E-08
positive regulation of multicellular organismal process (GO:0051240)	1783	133	72.6	+	1.83	5.58E-11	2.48E-08
neuron development (GO:0048666)	828	78	33.71	+	2.31	6.03E-11	2.60E-08
negative regulation of multicellular organismal process (GO:0051241)	1224	102	49.84	+	2.05	6.18E-11	2.59E-08
regulation of cell communication (GO:0010646)	3646	227	148.46	+	1.53	6.44E-11	2.63E-08

© 2020. Galkin G et al. Published online at http://www.aginganddisease.org/EN/10.14336/AD.2020.1202

regulation of signaling (GO:0023051)	3686	228	150.08	+	1.52	1.05E-10	4.20E-08
regulation of nervous system development (GO:0051960)	952	85	38.76	+	2.19	1.10E-10	4.27E-08
positive regulation of biological process (GO:0048518)	6278	346	255.62	+	1.35	1.14E-10	4.31E-08
signal transduction (GO:0007165)	5146	295	209.53	+	1.41	1.46E-10	5.40E-08
positive regulation of cellular process (GO:0048522)	5725	321	233.11	+	1.38	1.63E-10	5.90E-08
cell development (GO:0048468)	1632	123	66.45	+	1.85	1.91E-10	6.78E-08
central nervous system development (GO:0007417)	1001	87	40.76	+	2.13	1.97E-10	6.82E-08
positive regulation of developmental process (GO:0051094)	1401	110	57.05	+	1.93	2.23E-10	7.57E-08
regulation of transport (GO:0051049)	1906	137	77.61	+	1.77	3.01E-10	1.00E-07
neuron projection development (GO:0031175)	683	67	27.81	+	2.41	3.03E-10	9.88E-08
response to organic substance (GO:0010033)	3033	194	123.5	+	1.57	3.19E-10	1.02E-07
cellular response to endogenous stimulus (GO:0071495)	1224	99	49.84	+	1.99	4.47E-10	1.40E-07
localization (GO:0051179)	5802	321	236.24	+	1.36	6.92E-10	2.12E-07
cellular response to chemical stimulus (GO:0070887)	2961	189	120.56	+	1.57	7.59E-10	2.29E-07
negative regulation of biological process (GO:0048519)	5541	308	225.62	+	1.37	1.36E-09	4.03E-07
regulation of cell population proliferation (GO:0042127)	1656	121	67.43	+	1.79	1.39E-09	4.03E-07
negative regulation of developmental process (GO:0051093)	972	83	39.58	+	2.1	1.44E-09	4.11E-07
locomotion (GO:0040011)	1314	102	53.5	+	1.91	1.73E-09	4.85E-07
positive regulation of cell differentiation (GO:0045597)	1001	83	40.76	+	2.04	4.35E-09	1.20E-06
regulation of response to stimulus (GO:0048583)	4403	254	179.28	+	1.42	4.41E-09	1.19E-06
tissue development (GO:0009888)	1789	126	72.84	+	1.73	5.00E-09	1.33E-06
detection of chemical stimulus involved in sensory perception (GO:0050907)	483	0	19.67	-	< 0.01	6.92E-09	1.81E-06
cell fate commitment (GO:0045165)	252	34	10.26	+	3.31	8.81E-09	2.27E-06
embryonic organ development (GO:0048568)	447	48	18.2	+	2.64	9.85E-09	2.49E-06
cell migration (GO:0016477)	957	79	38.97	+	2.03	1.42E-08	3.53E-06
negative regulation of neurogenesis (GO:0050768)	292	36	11.89	+	3.03	2.66E-08	6.53E-06
neuron projection morphogenesis (GO:0048812)	494	50	20.11	+	2.49	2.84E-08	6.87E-06

© 2020. Galkin G et al. Published online at http://www.aginganddisease.org/EN/10.14336/AD.2020.1202

negative regulation of cell development (GO:0010721)	335	39	13.64	+	2.86	2.88E-08	6.85E-06
detection of chemical stimulus involved in sensory perception of smell (GO:0050911)	439	0	17.87	-	< 0.01	3.28E-08	7.71E-06
plasma membrane bounded cell projection morphogenesis (GO:0120039)	498	50	20.28	+	2.47	3.32E-08	7.68E-06
central nervous system neuron differentiation (GO:0021953)	190	28	7.74	+	3.62	3.42E-08	7.80E-06
cellular response to oxygen-containing compound (GO:1901701)	1080	85	43.97	+	1.93	3.58E-08	8.04E-06
cell projection morphogenesis (GO:0048858)	502	50	20.44	+	2.45	3.95E-08	8.76E-06
negative regulation of cell differentiation (GO:0045596)	715	63	29.11	+	2.16	4.74E-08	1.04E-05
regulation of signal transduction (GO:0009966)	3160	190	128.67	+	1.48	5.07E-08	1.09E-05
negative regulation of cellular process (GO:0048523)	4888	270	199.03	+	1.36	5.21E-08	1.11E-05
negative regulation of nervous system development (GO:0051961)	315	37	12.83	+	2.88	5.30E-08	1.11E-05
regulation of cellular component movement (GO:0051270)	1027	81	41.82	+	1.94	6.58E-08	1.36E-05
cell part morphogenesis (GO:0032990)	524	51	21.34	+	2.39	7.44E-08	1.52E-05
tube development (GO:0035295)	859	71	34.98	+	2.03	8.40E-08	1.70E-05
response to organonitrogen compound (GO:0010243)	1014	80	41.29	+	1.94	8.65E-08	1.73E-05
embryo development (GO:0009790)	1016	80	41.37	+	1.93	8.86E-08	1.75E-05
regulation of neuron differentiation (GO:0045664)	672	60	27.36	+	2.19	8.97E-08	1.75E-05
embryonic morphogenesis (GO:0048598)	581	54	23.66	+	2.28	1.04E-07	2.01E-05
cell-cell signaling (GO:0007267)	1175	88	47.84	+	1.84	1.25E-07	2.37E-05
movement of cell or subcellular component (GO:0006928)	1570	109	63.93	+	1.71	1.31E-07	2.46E-05
cell morphogenesis involved in neuron differentiation (GO:0048667)	445	45	18.12	+	2.48	1.68E-07	3.13E-05
regulation of anatomical structure morphogenesis (GO:0022603)	1107	84	45.07	+	1.86	1.87E-07	3.44E-05
sensory organ development (GO:0007423)	560	52	22.8	+	2.28	1.99E-07	3.61E-05
embryonic organ morphogenesis (GO:0048562)	305	35	12.42	+	2.82	2.02E-07	3.62E-05
localization of cell (GO:0051674)	1078	82	43.89	+	1.87	2.04E-07	3.62E-05
cell motility (GO:0048870)	1078	82	43.89	+	1.87	2.04E-07	3.58E-05
positive regulation of cell development (GO:0010720)	564	52	22.96	+	2.26	2.25E-07	3.90E-05

mesenchymal cell development (GO:0014031)	81	17	3.3	+	5.15	2.35E-07	4.03E-05
cellular response to drug (GO:0035690)	411	42	16.73	+	2.51	2.44E-07	4.14E-05
response to oxygen-containing compound (GO:1901700)	1609	110	65.51	+	1.68	2.51E-07	4.21E-05
response to drug (GO:0042493)	1030	79	41.94	+	1.88	2.62E-07	4.35E-05
sensory organ morphogenesis (GO:0090596)	268	32	10.91	+	2.93	3.01E-07	4.95E-05
regulation of ion transport (GO:0043269)	705	60	28.71	+	2.09	3.18E-07	5.18E-05
cell junction organization (GO:0034330)	490	47	19.95	+	2.36	3.49E-07	5.63E-05
response to organic cyclic compound (GO:0014070)	930	73	37.87	+	1.93	3.97E-07	6.33E-05
anatomical structure formation involved in morphogenesis (GO:0048646)	898	71	36.56	+	1.94	3.99E-07	6.31E-05
negative regulation of cellular component movement (GO:0051271)	315	35	12.83	+	2.73	4.04E-07	6.33E-05
cellular component morphogenesis (GO:0032989)	613	54	24.96	+	2.16	4.44E-07	6.87E-05
cell morphogenesis (GO:0000902)	729	61	29.68	+	2.06	4.75E-07	7.28E-05
neural crest cell differentiation (GO:0014033)	86	17	3.5	+	4.85	4.91E-07	7.47E-05
axon development (GO:0061564)	416	42	16.94	+	2.48	5.02E-07	7.56E-05
regulation of locomotion (GO:0040012)	982	75	39.98	+	1.88	5.54E-07	8.26E-05
response to growth factor (GO:0070848)	531	49	21.62	+	2.27	5.67E-07	8.38E-05
positive regulation of cell population proliferation (GO:0008284)	925	72	37.66	+	1.91	5.81E-07	8.50E-05
neural crest cell development (GO:0014032)	77	16	3.14	+	5.1	5.94E-07	8.62E-05
cellular component organization (GO:0016043)	5536	293	225.41	+	1.3	6.04E-07	8.68E-05
ossification (GO:0001503)	264	31	10.75	+	2.88	6.37E-07	9.08E-05
response to nitrogen compound (GO:1901698)	1098	81	44.71	+	1.81	8.74E-07	1.23E-04
pattern specification process (GO:0007389)	432	42	17.59	+	2.39	8.82E-07	1.23E-04
respiratory system development (GO:0060541)	200	26	8.14	+	3.19	9.22E-07	1.28E-04
stem cell development (GO:0048864)	81	16	3.3	+	4.85	1.07E-06	1.47E-04
response to chemical (GO:0042221)	4520	246	184.04	+	1.34	1.08E-06	1.47E-04
neural crest cell migration (GO:0001755)	52	13	2.12	+	6.14	1.15E-06	1.55E-04
circulatory system development (GO:0072359)	876	68	35.67	+	1.91	1.18E-06	1.59E-04
response to alcohol (GO:0097305)	245	29	9.98	+	2.91	1.26E-06	1.67E-04
forebrain development (GO:0030900)	404	40	16.45	+	2.43	1.26E-06	1.67E-04
positive regulation of neurogenesis (GO:0050769)	488	45	19.87	+	2.26	1.32E-06	1.72E-04
brain development (GO:0007420)	757	61	30.82	+	1.98	1.34E-06	1.74E-04

chemical synaptic transmission (GO:0007268)	426	41	17.35	+	2.36	1.47E-06	1.89E-04
anterograde trans-synaptic signaling (GO:0098916)	426	41	17.35	+	2.36	1.47E-06	1.87E-04
cellular response to growth factor stimulus (GO:0071363)	502	46	20.44	+	2.25	1.61E-06	2.04E-04
negative regulation of response to stimulus (GO:0048585)	1836	118	74.76	+	1.58	1.66E-06	2.08E-04
synapse organization (GO:0050808)	293	32	11.93	+	2.68	1.76E-06	2.20E-04
modulation of chemical synaptic transmission (GO:0050804)	459	43	18.69	+	2.3	1.79E-06	2.22E-04
positive regulation of nervous system development (GO:0051962)	556	49	22.64	+	2.16	1.80E-06	2.21E-04
G protein-coupled receptor signaling pathway, coupled to cyclic nucleotide second messenger (GO:0007187)	250	29	10.18	+	2.85	1.82E-06	2.21E-04
regulation of cell motility (GO:2000145)	942	71	38.36	+	1.85	1.82E-06	2.20E-04
plasma membrane bounded cell projection organization (GO:0120036)	1126	81	45.85	+	1.77	1.84E-06	2.21E-04
regulation of trans-synaptic signaling (GO:0099177)	460	43	18.73	+	2.3	1.86E-06	2.21E-04
regionalization (GO:0003002)	336	35	13.68	+	2.56	1.90E-06	2.25E-04
muscle system process (GO:0003012)	295	32	12.01	+	2.66	2.01E-06	2.36E-04
negative regulation of cell population proliferation (GO:0008285)	698	57	28.42	+	2.01	2.12E-06	2.47E-04
positive regulation of cell communication (GO:0010647)	1943	123	79.11	+	1.55	2.17E-06	2.51E-04
regulation of system process (GO:0044057)	592	51	24.1	+	2.12	2.19E-06	2.52E-04
regulation of ossification (GO:0030278)	197	25	8.02	+	3.12	2.20E-06	2.51E-04
trans-synaptic signaling (GO:0099537)	445	42	18.12	+	2.32	2.23E-06	2.52E-04
negative regulation of developmental growth (GO:0048640)	109	18	4.44	+	4.06	2.28E-06	2.56E-04
axonogenesis (GO:0007409)	380	38	15.47	+	2.46	2.29E-06	2.56E-04
cell junction assembly (GO:0034329)	268	30	10.91	+	2.75	2.35E-06	2.60E-04
circulatory system process (GO:0003013)	402	39	16.37	+	2.38	2.38E-06	2.62E-04
regulation of hormone levels (GO:0010817)	546	48	22.23	+	2.16	2.50E-06	2.74E-04
chemotaxis (GO:0006935)	547	48	22.27	+	2.16	2.57E-06	2.79E-04
diencephalon development (GO:0021536)	77	15	3.14	+	4.78	2.68E-06	2.89E-04
cell projection organization (GO:0030030)	1171	83	47.68	+	1.74	2.69E-06	2.88E-04
taxis (GO:0042330)	549	48	22.35	+	2.15	2.71E-06	2.89E-04

synaptic signaling (GO:0099536)	470	43	19.14	+	2.25	2.74E-06	2.89E-04
locomotory behavior (GO:0007626)	200	25	8.14	+	3.07	2.81E-06	2.95E-04
regulation of cell migration (GO:0030334)	879	67	35.79	+	1.87	2.82E-06	2.94E-04
cellular component organization or biogenesis (GO:0071840)	5754	298	234.29	+	1.27	3.22E-06	3.34E-04
cellular response to organonitrogen compound (GO:0071417)	606	51	24.67	+	2.07	3.23E-06	3.33E-04
blood circulation (GO:0008015)	392	38	15.96	+	2.38	3.33E-06	3.41E-04
negative regulation of locomotion (GO:0040013)	313	33	12.74	+	2.59	3.36E-06	3.41E-04
system process (GO:0003008)	1989	124	80.99	+	1.53	3.77E-06	3.80E-04
head development (GO:0060322)	802	62	32.66	+	1.9	4.01E-06	4.02E-04
positive regulation of signaling (GO:0023056)	1951	122	79.44	+	1.54	4.10E-06	4.09E-04
positive regulation of gene expression (GO:0010628)	2038	126	82.98	+	1.52	4.73E-06	4.69E-04
positive regulation of transcription, DNA-templated (GO:0045893)	1560	102	63.52	+	1.61	4.95E-06	4.88E-04
negative regulation of transport (GO:0051051)	500	44	20.36	+	2.16	5.43E-06	5.31E-04
detection of stimulus involved in sensory perception (GO:0050906)	546	4	22.23	-	0.18	5.79E-06	5.64E-04
regulation of molecular function (GO:0065009)	3073	175	125.12	+	1.4	6.02E-06	5.82E-04
cell morphogenesis involved in differentiation (GO:0000904)	565	48	23.01	+	2.09	6.21E-06	5.97E-04
positive regulation of signal transduction (GO:0009967)	1709	109	69.59	+	1.57	6.54E-06	6.25E-04
positive regulation of molecular function (GO:0044093)	1832	115	74.59	+	1.54	7.05E-06	6.70E-04
response to toxic substance (GO:0009636)	520	45	21.17	+	2.13	7.41E-06	7.00E-04
regulation of anatomical structure size (GO:0090066)	521	45	21.21	+	2.12	7.61E-06	7.14E-04
chemical homeostasis (GO:0048878)	1131	79	46.05	+	1.72	8.11E-06	7.57E-04
RNA processing (GO:0006396)	911	13	37.09	-	0.35	8.20E-06	7.61E-04
regulation of neurotransmitter levels (GO:0001505)	359	35	14.62	+	2.39	8.29E-06	7.65E-04
cellular response to nitrogen compound (GO:1901699)	663	53	27	+	1.96	8.41E-06	7.72E-04
ion homeostasis (GO:0050801)	798	61	32.49	+	1.88	8.41E-06	7.67E-04
lung development (GO:0030324)	173	22	7.04	+	3.12	8.56E-06	7.76E-04
growth (GO:0040007)	405	38	16.49	+	2.3	8.86E-06	7.99E-04
regulation of secretion (GO:0051046)	837	63	34.08	+	1.85	8.95E-06	8.02E-04

© 2020. Galkin G et al. Published online at http://www.aginganddisease.org/EN/10.14336/AD.2020.1202

ameboidal-type cell migration (GO:0001667)	174	22	7.08	+	3.11	9.29E-06	8.29E-04
regulation of cellular localization (GO:0060341)	1026	73	41.78	+	1.75	9.56E-06	8.48E-04
adult behavior (GO:0030534)	148	20	6.03	+	3.32	9.76E-06	8.61E-04
regulation of response to external stimulus (GO:0032101)	1196	82	48.7	+	1.68	9.89E-06	8.68E-04
ear development (GO:0043583)	231	26	9.41	+	2.76	9.97E-06	8.70E-04
camera-type eye morphogenesis (GO:0048593)	124	18	5.05	+	3.57	1.13E-05	9.80E-04
regulation of blood vessel diameter (GO:0097746)	137	19	5.58	+	3.41	1.16E-05	1.00E-03
regulation of tube diameter (GO:0035296)	137	19	5.58	+	3.41	1.16E-05	9.99E-04
mesenchyme development (GO:0060485)	219	25	8.92	+	2.8	1.18E-05	1.01E-03
respiratory tube development (GO:0030323)	177	22	7.21	+	3.05	1.18E-05	1.01E-03
homeostatic process (GO:0042592)	1668	106	67.92	+	1.56	1.24E-05	1.05E-03
cellular response to alcohol (GO:0097306)	89	15	3.62	+	4.14	1.26E-05	1.06E-03
regulation of tube size (GO:0035150)	138	19	5.62	+	3.38	1.28E-05	1.07E-03
neuromuscular process (GO:0050905)	113	17	4.6	+	3.69	1.30E-05	1.08E-03
synapse assembly (GO:0007416)	101	16	4.11	+	3.89	1.31E-05	1.08E-03
response to lipid (GO:0033993)	869	64	35.38	+	1.81	1.32E-05	1.08E-03
cellular response to organic cyclic compound (GO:0071407)	550	46	22.39	+	2.05	1.32E-05	1.08E-03
heart morphogenesis (GO:0003007)	250	27	10.18	+	2.65	1.34E-05	1.09E-03
negative regulation of growth (GO:0045926)	250	27	10.18	+	2.65	1.34E-05	1.08E-03
developmental growth (GO:0048589)	399	37	16.25	+	2.28	1.36E-05	1.10E-03
positive regulation of macromolecule metabolic process (GO:0010604)	3417	189	139.13	+	1.36	1.43E-05	1.15E-03
mesenchymal cell differentiation (GO:0048762)	153	20	6.23	+	3.21	1.51E-05	1.21E-03
response to hormone (GO:0009725)	924	67	37.62	+	1.78	1.56E-05	1.24E-03
detection of chemical stimulus (GO:0009593)	519	4	21.13	-	0.19	1.69E-05	1.33E-03
limb development (GO:0060173)	182	22	7.41	+	2.97	1.75E-05	1.38E-03
appendage development (GO:0048736)	182	22	7.41	+	2.97	1.75E-05	1.37E-03
sensory perception of smell (GO:0007608)	468	3	19.06	-	0.16	1.77E-05	1.38E-03
negative regulation of neuron differentiation (GO:0045665)	225	25	9.16	+	2.73	1.79E-05	1.39E-03
tube morphogenesis (GO:0035239)	659	52	26.83	+	1.94	1.80E-05	1.39E-03
positive regulation of nitrogen compound metabolic process (GO:0051173)	3239	180	131.88	+	1.36	1.81E-05	1.39E-03

© 2020. Galkin G et al. Published online at http://www.aginganddisease.org/EN/10.14336/AD.2020.1202

vascular process in circulatory system (GO:0003018)	169	21	6.88	+	3.05	1.86E-05	1.42E-03
negative regulation of cell communication (GO:0010648)	1428	93	58.14	+	1.6	1.88E-05	1.43E-03
negative regulation of signaling (GO:0023057)	1432	93	58.31	+	1.59	1.94E-05	1.46E-03
RNA metabolic process (GO:0016070)	1702	37	69.3	-	0.53	1.94E-05	1.46E-03
regulation of ion transmembrane transport (GO:0034765)	490	42	19.95	+	2.11	1.97E-05	1.47E-03
regulation of transcription by RNA polymerase II (GO:0006357)	2264	134	92.18	+	1.45	2.04E-05	1.52E-03
positive regulation of nucleic acid-templated transcription (GO:1903508)	1658	104	67.51	+	1.54	2.16E-05	1.60E-03
positive regulation of RNA biosynthetic process (GO:1902680)	1659	104	67.55	+	1.54	2.18E-05	1.61E-03
stem cell differentiation (GO:0048863)	158	20	6.43	+	3.11	2.30E-05	1.69E-03
striated muscle tissue development (GO:0014706)	287	29	11.69	+	2.48	2.35E-05	1.72E-03
adenylate cyclase-modulating G protein-coupled receptor signaling pathway (GO:0007188)	215	24	8.75	+	2.74	2.45E-05	1.78E-03
cyclic-nucleotide-mediated signaling (GO:0019935)	173	21	7.04	+	2.98	2.56E-05	1.85E-03
cardiovascular system development (GO:0072358)	532	44	21.66	+	2.03	2.62E-05	1.89E-03
regulation of cell death (GO:0010941)	1706	106	69.46	+	1.53	2.72E-05	1.95E-03
skeletal system development (GO:0001501)	502	42	20.44	+	2.05	2.76E-05	1.98E-03
regulation of cellular component organization (GO:0051128)	2428	141	98.86	+	1.43	2.83E-05	2.01E-03
epithelium development (GO:0060429)	1121	76	45.64	+	1.67	3.07E-05	2.18E-03
inner ear development (GO:0048839)	204	23	8.31	+	2.77	3.11E-05	2.20E-03
positive regulation of cellular metabolic process (GO:0031325)	3405	186	138.64	+	1.34	3.28E-05	2.31E-03
regulation of axon guidance (GO:1902667)	43	10	1.75	+	5.71	3.37E-05	2.36E-03
regulation of axonogenesis (GO:0050770)	191	22	7.78	+	2.83	3.43E-05	2.39E-03
regulation of peptide hormone secretion (GO:0090276)	220	24	8.96	+	2.68	3.43E-05	2.38E-03
regulation of transmembrane transport (GO:0034762)	575	46	23.41	+	1.96	3.44E-05	2.38E-03
positive regulation of transcription by RNA polymerase II (GO:0045944)	1224	81	49.84	+	1.63	3.45E-05	2.37E-03
regulation of developmental growth (GO:0048638)	342	32	13.93	+	2.3	3.64E-05	2.49E-03
regulation of gliogenesis (GO:0014013)	124	17	5.05	+	3.37	3.78E-05	2.58E-03
ion transport (GO:0006811)	1342	87	54.64	+	1.59	3.84E-05	2.61E-03

© 2020. Galkin G et al. Published online at http://www.aginganddisease.org/EN/10.14336/AD.2020.1202

heart development (GO:0007507)	526	43	21.42	+	2.01	3.97E-05	2.69E-03
positive regulation of metabolic process (GO:0009893)	3716	200	151.31	+	1.32	4.01E-05	2.70E-03
axon guidance (GO:0007411)	265	27	10.79	+	2.5	4.22E-05	2.83E-03
eye morphogenesis (GO:0048592)	152	19	6.19	+	3.07	4.27E-05	2.85E-03
mRNA metabolic process (GO:0016071)	696	9	28.34	-	0.32	4.36E-05	2.90E-03
regulation of muscle organ development (GO:0048634)	139	18	5.66	+	3.18	4.45E-05	2.95E-03
ear morphogenesis (GO:0042471)	126	17	5.13	+	3.31	4.53E-05	2.99E-03
neuron projection guidance (GO:0097485)	268	27	10.91	+	2.47	4.80E-05	3.15E-03
negative regulation of axon guidance (GO:1902668)	27	8	1.1	+	7.28	4.88E-05	3.19E-03
response to auditory stimulus (GO:0010996)	27	8	1.1	+	7.28	4.88E-05	3.18E-03
positive regulation of response to stimulus (GO:0048584)	2458	141	100.08	+	1.41	4.98E-05	3.23E-03
cardiac chamber morphogenesis (GO:0003206)	128	17	5.21	+	3.26	5.41E-05	3.50E-03
regulation of neuron projection development (GO:0010975)	516	42	21.01	+	2	5.42E-05	3.49E-03
response to estradiol (GO:0032355)	143	18	5.82	+	3.09	6.21E-05	3.98E-03
intracellular signal transduction (GO:0035556)	1705	104	69.42	+	1.5	6.27E-05	4.00E-03
negative regulation of ion transport (GO:0043271)	157	19	6.39	+	2.97	6.34E-05	4.03E-03
adenylate cyclase-activating G protein-coupled receptor signaling pathway (GO:0007189)	130	17	5.29	+	3.21	6.44E-05	4.08E-03
anterior/posterior pattern specification (GO:0009952)	216	23	8.79	+	2.62	6.95E-05	4.38E-03
muscle tissue development (GO:0060537)	301	29	12.26	+	2.37	7.05E-05	4.43E-03
regulation of nitrogen compound metabolic process (GO:0051171)	5891	294	239.87	+	1.23	7.47E-05	4.68E-03
regulation of cation channel activity (GO:2001257)	188	21	7.65	+	2.74	7.65E-05	4.77E-03
inner ear morphogenesis (GO:0042472)	106	15	4.32	+	3.48	7.73E-05	4.80E-03
muscle contraction (GO:0006936)	246	25	10.02	+	2.5	8.72E-05	5.40E-03
multicellular organismal response to stress (GO:0033555)	71	12	2.89	+	4.15	8.96E-05	5.52E-03
vasculature development (GO:0001944)	522	42	21.25	+	1.98	9.25E-05	5.68E-03
regulation of membrane potential (GO:0042391)	441	37	17.96	+	2.06	1.01E-04	6.20E-03
regulation of cell morphogenesis involved in differentiation (GO:0010769)	313	29	12.74	+	2.28	1.02E-04	6.20E-03
regulation of primary metabolic process (GO:0080090)	6077	301	247.44	+	1.22	1.02E-04	6.18E-03

response to external stimulus (GO:0009605)	2477	140	100.86	+	1.39	1.04E-04	6.29E-03
cell surface receptor signaling pathway (GO:0007166)	2493	141	101.51	+	1.39	1.08E-04	6.50E-03
blood vessel development (GO:0001568)	499	40	20.32	+	1.97	1.09E-04	6.54E-03
negative regulation of response to external stimulus (GO:0032102)	565	44	23.01	+	1.91	1.10E-04	6.55E-03
tissue morphogenesis (GO:0048729)	568	44	23.13	+	1.9	1.15E-04	6.87E-03
regulation of secretion by cell (GO:1903530)	783	56	31.88	+	1.76	1.17E-04	6.97E-03
protein complex oligomerization (GO:0051259)	235	24	9.57	+	2.51	1.19E-04	7.04E-03
sensory perception of chemical stimulus (GO:0007606)	540	6	21.99	-	0.27	1.21E-04	7.11E-03
positive regulation of blood circulation (GO:1903524)	74	12	3.01	+	3.98	1.27E-04	7.46E-03
regulation of ion transmembrane transporter activity (GO:0032412)	272	26	11.08	+	2.35	1.30E-04	7.61E-03
endocrine system development (GO:0035270)	125	16	5.09	+	3.14	1.31E-04	7.64E-03
positive regulation of cellular biosynthetic process (GO:0031328)	2027	118	82.53	+	1.43	1.31E-04	7.63E-03
spinal cord development (GO:0021510)	112	15	4.56	+	3.29	1.34E-04	7.78E-03
regulation of cellular metabolic process (GO:0031323)	6284	309	255.87	+	1.21	1.38E-04	7.95E-03
regulation of apoptotic process (GO:0042981)	1557	95	63.4	+	1.5	1.57E-04	8.99E-03
regulation of metabolic process (GO:0019222)	6772	329	275.74	+	1.19	1.62E-04	9.28E-03
positive regulation of RNA metabolic process (GO:0051254)	1748	104	71.17	+	1.46	1.63E-04	9.28E-03
negative regulation of gliogenesis (GO:0014014)	43	9	1.75	+	5.14	1.67E-04	9.48E-03
pituitary gland development (GO:0021983)	43	9	1.75	+	5.14	1.67E-04	9.45E-03
positive regulation of locomotion (GO:0040017)	562	43	22.88	+	1.88	1.68E-04	9.50E-03
skeletal system morphogenesis (GO:0048705)	244	24	9.94	+	2.42	1.71E-04	9.62E-03
tissue regeneration (GO:0042246)	65	11	2.65	+	4.16	1.73E-04	9.68E-03
cardiac chamber development (GO:0003205)	171	19	6.96	+	2.73	1.76E-04	9.81E-03
cell-cell adhesion (GO:0098609)	505	40	20.56	+	1.95	1.79E-04	9.95E-03
regulation of blood circulation (GO:1903522)	290	27	11.81	+	2.29	1.79E-04	9.93E-03
regulation of insulin secretion (GO:0050796)	186	20	7.57	+	2.64	1.79E-04	9.90E-03
positive regulation of biosynthetic process (GO:0009891)	2059	119	83.84	+	1.42	1.83E-04	1.01E-02
regulation of cell morphogenesis (GO:0022604)	512	40	20.85	+	1.92	2.01E-04	1.10E-02

regulation of cytosolic calcium ion concentration (GO:0051480)	342	30	13.93	+	2.15	2.02E-04	1.10E-02
response to wounding (GO:0009611)	579	44	23.58	+	1.87	2.03E-04	1.10E-02
regulation of synaptic vesicle cycle (GO:0098693)	118	15	4.8	+	3.12	2.25E-04	1.22E-02
regulation of programmed cell death (GO:0043067)	1579	95	64.29	+	1.48	2.28E-04	1.23E-02
rhythmic behavior (GO:0007622)	26	7	1.06	+	6.61	2.40E-04	1.29E-02
regulation of macromolecule metabolic process (GO:0060255)	6215	304	253.06	+	1.2	2.42E-04	1.30E-02
regulation of cell projection organization (GO:0031344)	700	50	28.5	+	1.75	2.43E-04	1.30E-02
positive regulation of macromolecule biosynthetic process (GO:0010557)	1934	112	78.75	+	1.42	2.48E-04	1.32E-02
regulation of retinal ganglion cell axon guidance (GO:0090259)	5	4	0.2	+	19.65	2.50E-04	1.33E-02
epoxide metabolic process (GO:0097176)	5	4	0.2	+	19.65	2.50E-04	1.33E-02
regulation of transcription, DNA-templated (GO:0006355)	3469	183	141.25	+	1.3	2.58E-04	1.36E-02
regulation of transmembrane transporter activity (GO:0022898)	281	26	11.44	+	2.27	2.59E-04	1.36E-02
negative regulation of chemotaxis (GO:0050922)	57	10	2.32	+	4.31	2.62E-04	1.37E-02
negative regulation of cell motility (GO:2000146)	282	26	11.48	+	2.26	2.67E-04	1.40E-02
positive regulation of neuron differentiation (GO:0045666)	382	32	15.55	+	2.06	2.69E-04	1.40E-02
response to abiotic stimulus (GO:0009628)	1165	74	47.44	+	1.56	2.76E-04	1.43E-02
regulation of nucleic acid-templated transcription (GO:1903506)	3536	186	143.98	+	1.29	2.84E-04	1.47E-02
cAMP-mediated signaling (GO:0019933)	149	17	6.07	+	2.8	2.86E-04	1.48E-02
regulation of RNA biosynthetic process (GO:2001141)	3541	186	144.18	+	1.29	2.88E-04	1.48E-02
positive regulation of nucleobase-containing compound metabolic process (GO:0045935)	1914	111	77.93	+	1.42	2.90E-04	1.49E-02
regulation of striated muscle tissue development (GO:0016202)	135	16	5.5	+	2.91	2.90E-04	1.49E-02
cellular chemical homeostasis (GO:0055082)	762	53	31.03	+	1.71	2.99E-04	1.52E-02
inorganic ion homeostasis (GO:0098771)	726	51	29.56	+	1.73	3.11E-04	1.58E-02
ncRNA metabolic process (GO:0034660)	515	6	20.97	-	0.29	3.24E-04	1.64E-02
regulation of plasma membrane bounded cell projection organization (GO:0120035)	691	49	28.14	+	1.74	3.26E-04	1.65E-02
negative regulation of cell migration (GO:0030336)	267	25	10.87	+	2.3	3.27E-04	1.65E-02

© 2020. Galkin G et al. Published online at http://www.aginganddisease.org/EN/10.14336/AD.2020.1202

central nervous system neuron development (GO:0021954)	83	12	3.38	+	3.55	3.30E-04	1.66E-02
wound healing (GO:0042060)	485	38	19.75	+	1.92	3.31E-04	1.66E-02
export from cell (GO:0140352)	1049	68	42.71	+	1.59	3.33E-04	1.66E-02
negative regulation of axonogenesis (GO:0050771)	71	11	2.89	+	3.8	3.42E-04	1.70E-02
second-messenger-mediated signaling (GO:0019932)	339	29	13.8	+	2.1	3.49E-04	1.73E-02
negative regulation of neuron projection development (GO:0010977)	152	17	6.19	+	2.75	3.53E-04	1.74E-02
regulation of muscle tissue development (GO:1901861)	138	16	5.62	+	2.85	3.63E-04	1.79E-02
dendrite morphogenesis (GO:0048813)	60	10	2.44	+	4.09	3.77E-04	1.85E-02
negative regulation of multicellular organism growth (GO:0040015)	12	5	0.49	+	10.23	3.78E-04	1.85E-02
cellular homeostasis (GO:0019725)	924	61	37.62	+	1.62	3.86E-04	1.88E-02
cellular response to lipid (GO:0071396)	529	40	21.54	+	1.86	3.88E-04	1.89E-02
cation homeostasis (GO:0055080)	714	50	29.07	+	1.72	3.96E-04	1.92E-02
regulation of hormone secretion (GO:0046883)	274	25	11.16	+	2.24	3.99E-04	1.93E-02
regulation of transporter activity (GO:0032409)	293	26	11.93	+	2.18	4.00E-04	1.93E-02
positive regulation of catalytic activity (GO:0043085)	1477	89	60.14	+	1.48	4.09E-04	1.97E-02
outflow tract morphogenesis (GO:0003151)	73	11	2.97	+	3.7	4.23E-04	2.03E-02
morphogenesis of an epithelium (GO:0002009)	440	35	17.92	+	1.95	4.25E-04	2.03E-02
blood vessel morphogenesis (GO:0048514)	410	33	16.69	+	1.98	4.36E-04	2.08E-02
gliogenesis (GO:0042063)	230	22	9.37	+	2.35	4.36E-04	2.07E-02
negative regulation of cell morphogenesis involved in differentiation (GO:0010771)	99	13	4.03	+	3.22	4.38E-04	2.07E-02
rhythmic process (GO:0048511)	277	25	11.28	+	2.22	4.42E-04	2.09E-02
bone mineralization (GO:0030282)	50	9	2.04	+	4.42	4.47E-04	2.10E-02
regulation of growth (GO:0040008)	684	48	27.85	+	1.72	4.52E-04	2.12E-02
cardiac septum morphogenesis (GO:0060411)	74	11	3.01	+	3.65	4.68E-04	2.19E-02
response to steroid hormone (GO:0048545)	329	28	13.4	+	2.09	4.80E-04	2.24E-02
cognition (GO:0050890)	307	27	12.5	+	2.16	4.81E-04	2.24E-02
small molecule metabolic process (GO:0044281)	1718	100	69.95	+	1.43	4.91E-04	2.28E-02
cellular ion homeostasis (GO:0006873)	660	47	26.87	+	1.75	4.91E-04	2.27E-02
regulation of nucleobase-containing compound metabolic process (GO:0019219)	4065	208	165.52	+	1.26	4.95E-04	2.28E-02

© 2020. Galkin G et al. Published online at http://www.aginganddisease.org/EN/10.14336/AD.2020.1202

embryonic limb morphogenesis (GO:0030326)	128	15	5.21	+	2.88	4.95E-04	2.28E-02
embryonic appendage morphogenesis (GO:0035113)	128	15	5.21	+	2.88	4.95E-04	2.27E-02
regulation of animal organ morphogenesis (GO:2000027)	260	24	10.59	+	2.27	4.97E-04	2.27E-02
activation of adenylate cyclase activity (GO:0007190)	40	8	1.63	+	4.91	5.00E-04	2.28E-02
negative regulation of signal transduction (GO:0009968)	1284	79	52.28	+	1.51	5.00E-04	2.27E-02
porphyrin-containing compound metabolic process (GO:0006778)	51	9	2.08	+	4.33	5.08E-04	2.30E-02
intermediate filament-based process (GO:0045103)	51	9	2.08	+	4.33	5.08E-04	2.29E-02
epithelial tube morphogenesis (GO:0060562)	311	27	12.66	+	2.13	5.26E-04	2.37E-02
transmembrane receptor protein serine/threonine kinase signaling pathway (GO:0007178)	198	20	8.06	+	2.48	5.38E-04	2.42E-02
gene expression (GO:0010467)	2043	54	83.19	-	0.65	5.72E-04	2.56E-02
regulation of bone mineralization (GO:0030500)	76	11	3.09	+	3.55	5.73E-04	2.56E-02
metal ion homeostasis (GO:0055065)	634	45	25.81	+	1.74	5.73E-04	2.55E-02
skeletal muscle tissue development (GO:0007519)	130	15	5.29	+	2.83	5.73E-04	2.55E-02
smooth muscle contraction (GO:0006939)	52	9	2.12	+	4.25	5.75E-04	2.55E-02
feeding behavior (GO:0007631)	89	12	3.62	+	3.31	5.81E-04	2.57E-02
response to ketone (GO:1901654)	201	20	8.18	+	2.44	5.93E-04	2.62E-02
response to pain (GO:0048265)	31	7	1.26	+	5.55	5.98E-04	2.63E-02
positive regulation of cellular component movement (GO:0051272)	563	41	22.92	+	1.79	6.05E-04	2.65E-02
cellular response to toxic substance (GO:0097237)	220	21	8.96	+	2.34	6.08E-04	2.66E-02
enzyme linked receptor protein signaling pathway (GO:0007167)	726	50	29.56	+	1.69	6.19E-04	2.70E-02
camera-type eye development (GO:0043010)	317	27	12.91	+	2.09	6.21E-04	2.70E-02
regulation of mitochondrial depolarization (GO:0051900)	22	6	0.9	+	6.7	6.34E-04	2.75E-02
positive regulation of hydrolase activity (GO:0051345)	770	52	31.35	+	1.66	6.47E-04	2.80E-02
skeletal muscle cell differentiation (GO:0035914)	53	9	2.16	+	4.17	6.50E-04	2.80E-02
regeneration (GO:0031099)	162	17	6.6	+	2.58	6.85E-04	2.95E-02
regulation of RNA metabolic process (GO:0051252)	3807	195	155.01	+	1.26	7.01E-04	3.01E-02

© 2020. Galkin G et al. Published online at http://www.aginganddisease.org/EN/10.14336/AD.2020.1202

porphyrin-containing compound biosynthetic process (GO:0006779)	32	7	1.3	+	5.37	7.04E-04	3.01E-02
transport (GO:0006810)	4550	228	185.26	+	1.23	7.13E-04	3.04E-02
cellular response to BMP stimulus (GO:0071773)	105	13	4.28	+	3.04	7.24E-04	3.08E-02
response to BMP (GO:0071772)	105	13	4.28	+	3.04	7.24E-04	3.07E-02
positive regulation of cell motility (GO:2000147)	547	40	22.27	+	1.8	7.24E-04	3.07E-02
developmental growth involved in morphogenesis (GO:0060560)	119	14	4.85	+	2.89	7.27E-04	3.07E-02
regulation of muscle contraction (GO:0006937)	163	17	6.64	+	2.56	7.30E-04	3.07E-02
biological adhesion (GO:0022610)	930	60	37.87	+	1.58	7.52E-04	3.16E-02
regulation of voltage-gated calcium channel activity (GO:1901385)	43	8	1.75	+	4.57	7.60E-04	3.19E-02
connective tissue development (GO:0061448)	225	21	9.16	+	2.29	7.63E-04	3.19E-02
cellular response to prostaglandin stimulus (GO:0071379)	23	6	0.94	+	6.41	7.73E-04	3.22E-02
response to stress (GO:0006950)	3635	187	148.01	+	1.26	7.83E-04	3.25E-02
mRNA processing (GO:0006397)	477	6	19.42	-	0.31	7.91E-04	3.28E-02
regulation of gene expression (GO:0010468)	4494	225	182.98	+	1.23	7.91E-04	3.27E-02
gland development (GO:0048732)	421	33	17.14	+	1.93	8.01E-04	3.30E-02
cellular response to nutrient (GO:0031670)	67	10	2.73	+	3.67	8.15E-04	3.35E-02
response to ethanol (GO:0045471)	135	15	5.5	+	2.73	8.17E-04	3.35E-02
tetrapyrrole biosynthetic process (GO:0033014)	33	7	1.34	+	5.21	8.25E-04	3.38E-02
response to prostaglandin (GO:0034694)	33	7	1.34	+	5.21	8.25E-04	3.37E-02
postsynapse organization (GO:0099173)	93	12	3.79	+	3.17	8.26E-04	3.36E-02
regulation of integrin activation (GO:0033623)	15	5	0.61	+	8.19	8.60E-04	3.49E-02
negative regulation of glial cell proliferation (GO:0060253)	15	5	0.61	+	8.19	8.60E-04	3.48E-02
mechanosensory behavior (GO:0007638)	15	5	0.61	+	8.19	8.60E-04	3.48E-02
regulation of cardiac muscle cell proliferation (GO:0060043)	44	8	1.79	+	4.47	8.68E-04	3.50E-02
limb morphogenesis (GO:0035108)	151	16	6.15	+	2.6	8.86E-04	3.56E-02
appendage morphogenesis (GO:0035107)	151	16	6.15	+	2.6	8.86E-04	3.55E-02
neuron fate commitment (GO:0048663)	68	10	2.77	+	3.61	9.03E-04	3.61E-02
nucleic acid metabolic process (GO:0090304)	2303	64	93.77	-	0.68	9.05E-04	3.61E-02
cellular metal ion homeostasis (GO:0006875)	570	41	23.21	+	1.77	9.15E-04	3.64E-02
response to catecholamine (GO:0071869)	108	13	4.4	+	2.96	9.17E-04	3.64E-02

© 2020. Galkin G et al. Published online at http://www.aginganddisease.org/EN/10.14336/AD.2020.1202

response to monoamine (GO:0071867)	108	13	4.4	+	2.96	9.17E-04	3.63E-02
trabecula formation (GO:0060343)	24	6	0.98	+	6.14	9.35E-04	3.69E-02
phosphorus metabolic process (GO:0006793)	2177	120	88.64	+	1.35	9.43E-04	3.71E-02
neuron fate specification (GO:0048665)	34	7	1.38	+	5.06	9.62E-04	3.78E-02
regulation of inflammatory response (GO:0050727)	537	39	21.87	+	1.78	9.63E-04	3.78E-02
segmentation (GO:0035282)	95	12	3.87	+	3.1	9.77E-04	3.82E-02
response to nutrient levels (GO:0031667)	538	39	21.91	+	1.78	9.78E-04	3.82E-02
reproductive structure development (GO:0048608)	432	33	17.59	+	1.88	9.81E-04	3.82E-02
regulation of epithelial cell proliferation (GO:0050678)	343	28	13.97	+	2	9.85E-04	3.83E-02
lung morphogenesis (GO:0060425)	45	8	1.83	+	4.37	9.88E-04	3.83E-02
regulation of monooxygenase activity (GO:0032768)	57	9	2.32	+	3.88	1.03E-03	3.99E-02
positive regulation of blood vessel diameter (GO:0097755)	57	9	2.32	+	3.88	1.03E-03	3.98E-02
neuron migration (GO:0001764)	124	14	5.05	+	2.77	1.05E-03	4.02E-02
regulation of neurotransmitter secretion (GO:0046928)	110	13	4.48	+	2.9	1.07E-03	4.10E-02
skeletal muscle organ development (GO:0060538)	139	15	5.66	+	2.65	1.07E-03	4.10E-02
establishment of localization (GO:0051234)	4680	232	190.56	+	1.22	1.09E-03	4.16E-02
cardiac muscle tissue regeneration (GO:0061026)	3	3	0.12	+	24.56	1.09E-03	4.16E-02
neuromuscular process controlling posture (GO:0050884)	16	5	0.65	+	7.67	1.09E-03	4.15E-02
coronary vasculature morphogenesis (GO:0060977)	16	5	0.65	+	7.67	1.09E-03	4.14E-02
response to ATP (GO:0033198)	35	7	1.43	+	4.91	1.12E-03	4.22E-02
response to progesterone (GO:0032570)	46	8	1.87	+	4.27	1.12E-03	4.23E-02
coronary vasculature development (GO:0060976)	46	8	1.87	+	4.27	1.12E-03	4.22E-02
negative regulation of amine transport (GO:0051953)	25	6	1.02	+	5.89	1.12E-03	4.21E-02
negative regulation of axon extension involved in axon guidance (GO:0048843)	25	6	1.02	+	5.89	1.12E-03	4.20E-02
circadian behavior (GO:0048512)	25	6	1.02	+	5.89	1.12E-03	4.19E-02
response to prostaglandin E (GO:0034695)	25	6	1.02	+	5.89	1.12E-03	4.18E-02
glial cell differentiation (GO:0010001)	179	18	7.29	+	2.47	1.13E-03	4.22E-02
learning or memory (GO:0007611)	265	23	10.79	+	2.13	1.14E-03	4.22E-02

© 2020. Galkin G et al. Published online at http://www.aginganddisease.org/EN/10.14336/AD.2020.1202

cell differentiation in spinal cord (GO:0021515)	58	9	2.36	+	3.81	1.15E-03	4.26E-02
cardiac septum development (GO:0003279)	111	13	4.52	+	2.88	1.15E-03	4.26E-02
secretion (GO:0046903)	1119	69	45.56	+	1.51	1.16E-03	4.27E-02
muscle structure development (GO:0061061)	483	36	19.67	+	1.83	1.16E-03	4.28E-02
negative regulation of cell projection organization (GO:0031345)	181	18	7.37	+	2.44	1.20E-03	4.41E-02
regulation of synaptic transmission, glutamatergic (GO:0051966)	71	10	2.89	+	3.46	1.21E-03	4.44E-02
cell adhesion (GO:0007155)	924	59	37.62	+	1.57	1.29E-03	4.70E-02
response to purine-containing compound (GO:0014074)	157	16	6.39	+	2.5	1.29E-03	4.70E-02
regulation of cellular biosynthetic process (GO:0031326)	4217	211	171.71	+	1.23	1.30E-03	4.71E-02
response to organophosphorus (GO:0046683)	142	15	5.78	+	2.59	1.30E-03	4.72E-02
regulation of cytokine production (GO:0001817)	734	49	29.89	+	1.64	1.31E-03	4.73E-02
phosphate-containing compound metabolic process (GO:0006796)	2150	118	87.54	+	1.35	1.31E-03	4.75E-02
regulation of sprouting angiogenesis (GO:1903670)	72	10	2.93	+	3.41	1.33E-03	4.81E-02
adenylate cyclase-activating adrenergic receptor signaling pathway (GO:0071880)	26	6	1.06	+	5.67	1.34E-03	4.80E-02
cellular response to prostaglandin E stimulus (GO:0071380)	17	5	0.69	+	7.22	1.37E-03	4.91E-02
Analysis Type:	PANTHER Overrepresentation Test (Released 20200407)						
Annotation Version and Release Date:	GO Ontology database DOI: 10.5281/zenodo.3727280 Released 2020-03-23						
Analyzed List:	DeepMage_genes (Homo sapiens)						
Reference List:	Homo sapiens (all genes in database)						
Test Type:	FISHER						
Correction:	FDR						

Supplementary Table 4. DeepMAge outperformed both the 71 CpG and the 89 CpG clocks published by Hannum et al. in 2013. The baseline is defined as the mean age assignment.

GEO Accession	MAE, years				RMSE, years				
	71 CpG	89 CpG	DeepMAge	Baseline	71 CpG	89 CpG	DeepMAge	Baseline	N
GSE102177	3.87	7.59	1.98	2.22	4.56	8.03	2.23	2.63	36
GSE103911	16.31	31.25	7.43	8.24	20.44	33.99	9.16	10.66	65
GSE105123	4.4	16.53	2.32	1.14	5.78	17.1	2.87	1.34	107
GSE107459	5.7	19.46	2.23	3.63	7.09	20.51	2.79	4.38	127

© 2020. Galkin G et al. Published online at http://www.aginganddisease.org/EN/10.14336/AD.2020.1202

GSE107737	8.19	15.9	3.87	2.88	9.99	17.12	4.96	3.32	24
GSE112696	6.89	14.64	4.42	2.33	7.23	15.14	5.17	2.94	12
GSE34639	0.98	1	1.79	0.5	1.1	1.12	2.06	0.5	48
GSE59065	8.31	19.49	5.93	23.46	10.26	21.11	8.14	23.63	295
GSE61496	18.14	22.85	2.77	15.07	22.01	27.23	3.7	15.42	310
GSE87582	7.63	17.16	5.4	4.53	9.46	19.41	6.04	5.67	21
GSE87640	14.12	19.35	4.53	9.78	17.85	23.52	6.54	11.9	240
GSE98876	10.45	21.13	3.49	7.99	14.13	24.48	4.64	9.98	71
GSE99624	8.89	19.71	4.12	8.74	11.41	22.05	5.43	9.81	48
Total	11.05	19.54	3.97	18.62	15.12	22.96	5.75	21.35	1404

GEO = Gene Expression Omnibus; N = Number of samples in the corresponding GEO project; MAE = Mean absolute error; RMSE = Root mean square error.

Supplementary Table 5. Linear regression coefficients for the model produced by replicating Horvath's training protocol. Ranks are based on absolute coefficient value (higher importance features come first).

Rank	CpG_site	coefficient	Rank	CpG_site	coefficient	Rank	CpG_site	coefficient	Rank	CpG_site	coefficient
1	cg21801378	0.983653	31	cg19885761	0.355402	61	cg05675373	0.237629	91	cg07845392	0.176361
2	cg02479575	0.939384	32	cg04836038	0.354184	62	cg16168311	-0.233731	92	cg19759064	-0.175474
3	cg00059225	0.816305	33	cg27015931	-0.348080	63	cg14918082	0.233498	93	cg05724065	-0.175079
4	cg22736354	0.793852	34	cg00630583	-0.342940	64	cg11017269	-0.232564	94	cg08668790	0.172569
5	cg01580888	0.681322	35	cg17861230	0.340669	65	cg04587910	-0.231216	95	cg23320649	-0.172294
6	cg06493994	0.582153	36	cg17471102	-0.337370	66	cg02840794	-0.229842	96	cg25713185	0.170709
7	cg19761273	-0.568068	37	cg09118625	0.332719	67	cg06836772	-0.223745	97	cg17706173	0.169717
8	cg27225570	-0.566827	38	cg13663218	-0.329015	68	cg20392764	0.220477	98	cg15957394	0.169437
9	(Intercept)	-0.549722	39	cg11299964	-0.328907	69	cg19357849	-0.216828	99	cg15804973	-0.168001
10	cg09809672	-0.532471	40	cg22580512	-0.326412	70	cg15297650	-0.216779	100	cg12422450	0.167622
11	cg00343092	-0.500909	41	cg13921352	0.320154	71	cg11296937	-0.215436	101	cg07158339	-0.166068
12	cg04474832	-0.463950	42	cg18328933	-0.318381	72	cg04564646	-0.210781	102	cg12340144	-0.165421
13	cg14915263	-0.461811	43	cg07850604	0.309722	73	cg12261786	-0.210380	103	cg02654291	0.160027
14	cg00503840	0.455829	44	cg23124451	-0.309382	74	cg18055007	0.208290	104	cg01459453	-0.157035
15	cg04528819	0.446599	45	cg21256649	-0.308976	75	cg07388493	-0.206733	105	cg05492845	-0.152588
16	cg25256723	-0.440934	46	cg08468689	-0.307241	76	cg00168942	-0.205629	106	cg04983977	0.151666
17	cg16785344	-0.422005	47	cg21296230	0.299826	77	cg22947000	-0.203777	107	cg18440048	0.150148
18	cg26005082	0.418708	48	cg20125091	0.291461	78	cg20692569	0.200028	108	cg11554937	0.146277
19	cg24170090	-0.409602	49	cg13975369	0.289659	79	cg06615861	-0.199849	109	cg01919208	0.145546
20	cg08888956	-0.408093	50	cg13494498	0.280318	80	cg12437239	0.197764	110	cg11007423	0.143615
21	cg08209133	0.406863	51	cg18815943	0.277910	81	cg18008766	0.189601	111	cg00451635	-0.131398
22	cg02228185	-0.406239	52	cg06885782	-0.276366	82	cg11465372	0.188791	112	cg18628483	-0.130806
23	cg27320127	0.404825	53	cg14754581	0.275500	83	cg25148589	0.187226	113	cg24127874	0.130118
24	cg13500819	0.390582	54	cg11668844	0.264290	84	cg12467090	0.185320	114	cg26842024	0.128317
25	cg01511567	-0.390299	55	cg19945840	0.260856	85	cg15798153	0.184923	115	cg10822172	-0.128046
26	cg19722847	-0.387929	56	cg27544190	-0.258460	86	cg15836394	-0.183558	116	cg19046959	-0.125864
27	cg22809047	0.371073	57	cg14732136	-0.254872	87	cg17291001	0.182513	117	cg03330058	-0.125722
28	cg07408456	-0.362516	58	cg26614073	-0.253922	88	cg13899108	0.181476	118	cg26847866	-0.124492

SUPPLEMENTARY DATA

29	cg20295671	-0.361302	59	cg01007201	0.246585	89	cg10523019	0.179182	119	cg19560758	0.124417
30	cg01820374	-0.355981	60	cg18236477	0.244505	90	cg21232015	0.178062	120	cg09706243	-0.124360

Rank	CpG_site	coefficient	Rank	CpG_site	coefficient	Rank	CpG_site	coefficient	Rank	CpG_site	coefficient
121	cg13460409	0.120700	151	cg20761322	0.098329	181	cg24231716	0.073321	211	cg15410903	-0.052966
122	cg16363586	-0.119329	152	cg23749046	0.096361	182	cg02085507	0.072851	212	cg19904653	0.052474
123	cg17465304	0.118849	153	cg20240860	-0.094244	183	cg27210390	-0.072419	213	cg16714091	0.052409
124	cg02142461	0.118652	154	cg18691434	0.093386	184	cg20732137	0.072384	214	cg20419410	-0.052102
125	cg05671018	0.116750	155	cg25771195	0.092643	185	cg11051139	-0.070007	215	cg06288351	-0.052079
126	cg00433406	0.116722	156	cg27491887	0.092029	186	cg20143092	-0.066992	216	cg10648908	-0.051900
127	cg12024906	0.116126	157	cg23674788	0.089757	187	cg02196655	0.066945	217	cg23968383	0.051490
128	cg11896923	-0.115847	158	cg09238598	-0.089702	188	cg04425624	-0.065455	218	cg00563932	-0.050910
129	cg09667582	0.115700	159	cg00528967	-0.088380	189	cg16034652	0.064228	219	cg10681065	0.050569
130	cg14360917	0.115690	160	cg13697378	0.087401	190	cg08072716	0.064033	220	cg21432842	-0.050569
131	cg25762706	0.113164	161	cg15201877	0.086424	191	cg25020850	0.063756	221	cg05436231	0.050171
132	cg15538427	-0.112250	162	cg26203861	-0.085473	192	cg22971191	0.063552	222	cg15792367	-0.049766
133	cg17628717	0.111783	163	cg08317263	-0.085192	193	cg06238491	-0.063003	223	cg16529592	0.048889
134	cg15095327	0.111534	164	cg00689340	-0.083082	194	cg11618577	0.062549	224	cg02828104	-0.048105
135	cg16547529	0.111106	165	cg12782180	0.082635	195	cg26372517	0.062243	225	cg16330965	0.047424
136	cg09462576	-0.109677	166	cg15156836	-0.082545	196	cg18081258	-0.061605	226	cg17191178	0.047321
137	cg15013019	-0.108504	167	cg26673195	-0.082272	197	cg15563382	0.060942	227	cg11879514	0.046374
138	cg00240880	0.106645	168	cg02844545	0.081288	198	cg00187380	-0.060720	228	cg10322876	0.046245
139	cg16744741	-0.106124	169	cg23894058	0.080975	199	cg08032971	0.059374	229	cg25372195	0.045917
140	cg25459323	-0.105747	170	cg17655614	0.079729	200	cg02310296	0.058791	230	cg20969242	0.045905
141	cg19724470	-0.104476	171	cg22909609	-0.079372	201	cg23887396	-0.057687	231	cg13493001	0.045881
142	cg18303397	-0.103026	172	cg11393848	-0.078721	202	cg01600189	-0.056979	232	cg12830694	0.045029
143	cg26083396	-0.102282	173	cg10989517	0.077383	203	cg14709524	0.056268	233	cg03991512	-0.044471
144	cg12167564	0.102049	174	cg23303074	-0.076855	204	cg21426387	-0.056218	234	cg26845300	0.043818
145	cg07211259	-0.100932	175	cg11126134	0.076425	205	cg26394940	-0.055434	235	cg22472290	0.042385
146	cg00757070	-0.100919	176	cg21600563	-0.076349	206	cg20582779	0.055430	236	cg05358404	0.042356
147	cg19850406	0.100517	177	cg27016307	-0.075887	207	cg12289045	0.055241	237	cg08965235	-0.041639
148	cg24081819	-0.100099	178	cg13813391	0.075797	208	cg11819637	0.054954	238	cg10674793	0.041490
149	cg05749577	-0.099570	179	cg24847163	-0.075789	209	cg02489552	0.053384	239	cg10676060	0.041424
150	cg16913124	0.099268	180	cg09307279	-0.075082	210	cg19777783	0.053383	240	cg03019000	-0.041335

Rank	CpG_site	coefficient	Rank	CpG_site	coefficient	Rank	CpG_site	coefficient	Rank	CpG_site	coefficient
241	cg03929796	-0.041099	271	cg04304130	0.025783	301	cg19356189	-0.013159	331	cg03805684	0.004344
242	cg14166009	0.040661	272	cg23854009	0.025638	302	cg09195271	0.012800	332	cg26376809	-0.004212
243	cg22022041	0.040321	273	cg06736444	0.025217	303	cg02829654	0.012382	333	cg20436912	0.003965
244	cg06458239	0.039813	274	cg23881601	-0.025032	304	cg05294243	0.012290	334	cg06144905	0.003305
245	cg21289015	0.039448	275	cg20648149	0.024993	305	cg27169020	0.012111	335	cg21098323	0.002805
246	cg08090640	-0.038989	276	cg03943081	0.024911	306	cg18847227	0.011814	336	cg20775254	0.002624
247	cg15408407	-0.038133	277	cg12774845	-0.024566	307	cg22335340	-0.011134	337	cg07895149	-0.002459
248	cg18017908	-0.037211	278	cg13269407	-0.024451	308	cg21301148	-0.010720	338	cg05507459	-0.002235
249	cg11219178	-0.036830	279	cg15537850	-0.024411	309	cg26057752	0.010613	339	cg20747455	0.002161
250	cg06896207	0.036030	280	cg22799321	0.023824	310	cg21448423	-0.010174	340	cg09554443	0.002072

© 2020. Galkin G et al. Published online at http://www.aginganddisease.org/EN/10.14336/AD.2020.1202

251	cg20492912	0.036028	281	cg25282410	0.023754	311	cg12426141	-0.010024	341	cg10281002	0.001749
252	cg22941086	0.035732	282	cg08558340	0.023500	312	cg20387706	0.009523	342	cg16519742	0.001362
253	cg06268694	0.035268	283	cg04123409	-0.022247	313	cg03224418	-0.008908	343	cg21652958	0.001228
254	cg25004981	0.033099	284	cg11976790	0.020600	314	cg18279742	0.008791	344	cg08723608	0.000896
255	cg10919204	0.032924	285	cg19109050	0.019781	315	cg08861115	0.008731	345	cg11377136	0.000827
256	cg26538442	0.032287	286	cg16858125	0.018828	316	cg10249734	0.008316	346	cg00208830	0.000789
257	cg15780361	0.031666	287	cg01560871	-0.016904	317	cg10917602	-0.008023	347	cg08124399	0.000608
258	cg06630241	0.031412	288	cg12717203	-0.016663	318	cg10362475	0.007987	348	cg07549715	0.000344
259	cg08331960	-0.030947	289	cg08022502	-0.016501	319	cg14972143	0.007970			
260	cg21057046	-0.030385	290	cg16254309	0.016370	320	cg05113558	0.007859			
261	cg25221254	0.029982	291	cg04633513	0.016306	321	cg18902090	0.007801			
262	cg04765422	0.029879	292	cg25809905	-0.015482	322	cg00308665	-0.007369			
263	cg06971096	0.029082	293	cg24341129	0.015423	323	cg15379633	0.007226			
264	cg18678185	-0.028961	294	cg21663431	-0.015366	324	cg05420896	0.006647			
265	cg26976437	0.028689	295	cg25564800	-0.014755	325	cg13726191	-0.006321			
266	cg22215728	0.028028	296	cg01656853	0.014466	326	cg00565075	0.005736			
267	cg22805308	0.027331	297	cg13302154	-0.014253	327	cg19595170	-0.005258			
268	cg26297688	0.027140	298	cg01400401	0.014041	328	cg14576824	0.005170			
269	cg08822227	-0.027097	299	cg22511947	0.013973	329	cg01753375	-0.004934			
270	cg17832674	-0.026777	300	cg17453778	0.013909	330	cg09150232	-0.004447			

© 2020. Galkin G et al. Published online at http://www.aginganddisease.org/EN/10.14336/AD.2020.1202
